# TBR2 coordinates neurogenesis expansion and precise microcircuit organization via Protocadherin 19 in the mammalian cortex

**DOI:** 10.1038/s41467-019-11854-x

**Published:** 2019-09-02

**Authors:** Xiaohui Lv, Si-Qiang Ren, Xin-Jun Zhang, Zhongfu Shen, Tanay Ghosh, Anjin Xianyu, Peng Gao, Zhizhong Li, Susan Lin, Yang Yu, Qiangqiang Zhang, Matthias Groszer, Song-Hai Shi

**Affiliations:** 1 Developmental Biology Program, Sloan Kettering Institute, Memorial Sloan Kettering Cancer Center, 1275 York Avenue, New York, NY 10065 USA; 2 IDG/McGovern Institute for Brain Research, Tsinghua-Peking Joint Center for Life Sciences, Beijing Frontier Research Center of Biological Structures, School of Life Sciences, Tsinghua University, Beijing, 100084 China; 3 Inserm, UMR-S839, Sorbonne Université, Institut du Fer à Moulin, Paris, 75005 France; 4 Department of Clinical Neurosciences, Wellcome Trust-Medical Research Council- Cambridge Stem Cell Institute, University of Cambridge, Cambridge, CB2 0AH UK; 5 Graduate Program in Biophysics, Weill Cornell Medical College, 1300 York Avenue, New York, NY 10065 USA; 6 Graduate Program in Neuroscience, Weill Cornell Medical College, 1300 York Avenue, New York, NY 10065 USA

**Keywords:** Neurogenesis, Neural stem cells

## Abstract

Cerebral cortex expansion is a hallmark of mammalian brain evolution; yet, how increased neurogenesis is coordinated with structural and functional development remains largely unclear. The T-box protein TBR2/EOMES is preferentially enriched in intermediate progenitors and supports cortical neurogenesis expansion. Here we show that TBR2 regulates fine-scale spatial and circuit organization of excitatory neurons in addition to enhancing neurogenesis in the mouse cortex. TBR2 removal leads to a significant reduction in neuronal, but not glial, output of individual radial glial progenitors as revealed by mosaic analysis with double markers. Moreover, in the absence of TBR2, clonally related excitatory neurons become more laterally dispersed and their preferential synapse development is impaired. Interestingly, TBR2 directly regulates the expression of Protocadherin 19 (PCDH19), and simultaneous PCDH19 expression rescues neurogenesis and neuronal organization defects caused by TBR2 removal. Together, these results suggest that TBR2 coordinates neurogenesis expansion and precise microcircuit assembly via PCDH19 in the mammalian cortex.

## Abstract (web summary)

The T-box protein TBR2 is involved in cortical neurogenesis expansion during neurodevelopment and is preferentially enriched in intermediate progenitors. The authors show that TBR2 coordinates neurogenesis expansion and precise microcircuit assembly in the mouse cortex via PCDH19.

## Introduction

The mammalian cerebral cortex is a complex yet highly organized brain structure, consisting of six layers of neurons that form specific circuits to support all higher-order brain functions. The formation of the cerebral cortex depends on the orderly production of a large number of diverse neurons, which largely occurs during embryonic development. The vast majority of cortical excitatory neurons originate from radial glial cells, the predominant population of neural progenitor cells in the developing cortex^1–8^. During cortical development, radial glial progenitors (RGPs) progress through a cellular program of proliferation, neurogenesis, and gliogenesis^4,5,9^. At the early stage of cortical development (e.g., embryonic day (E) 10-11 in mice), RGPs predominantly undergo symmetric proliferative division to amplify themselves. As development proceeds, they switch to asymmetric neurogenic division to self-renew and, at the same time, to produce neurons either directly (direct neurogenesis) or indirectly via transit amplifying progenitors (TAPs) that divide mostly in the subventricular zone (SVZ) to give rise to neurons (indirect neurogenesis)^8,10–17^.

Indirect neurogenesis via TAPs greatly increases the number of neurons generated in the cortex. As a consequence, the abundance and diversity of TAPs have been linked to evolutionary expansion of the cortex with higher cognitive functions^10,12–14,18–23^. A major population of TAPs consists of intermediate progenitors (IPs) that preferentially express the T-box transcription factor TBR2/EOMES^16^. Upon generation by dividing RGPs at the VZ surface, TBR2^+^ IPs migrate to the SVZ and mainly divide symmetrically to produce neurons. Given the clear importance of IPs in cortical neurogenesis, extensive efforts have been made to elucidate the precise contribution of TBR2^+^ IPs to the process; yet, the results have been inconsistent. Some studies suggested that TBR2 and IPs in the SVZ predominantly contribute to superficial layer neuron production and supragranular layer expansion^23–25^. Related to this, TBR2^+^ IPs and indirect neurogenesis have been linked to distinct morphological and electrophysiological features of superficial layer neurons^26^. On the other hand, a few studies have shown that TBR2^+^ IPs contribute to neuron production across all layers^27–30^. In addition, TBR2 has been suggested to influence neuronal differentiation and laminar fate specification in the cortex^30^. Together, these observations indicate that the precise contribution of TBR2^+^ IPs to cortical neurogenesis, especially at the level of individual RGPs, remains nebulous. It calls for an in-depth quantitative clonal analysis of the exact contribution of TBR2^+^ IPs to the neuronal as well as glial output of individual RGPs in the developing cortex.

Notably, the cellular processes of neurogenesis and neuronal migration critically influence the spatial distribution and functional organization of cortical excitatory neurons. In particular, clonally related excitatory neurons originating from the same neurogenic RGP (e.g., labeled at E12-13 in mice) migrate along their mother radial glial fiber and form an ontogenetic radial neuronal cluster spanning across both deep and superficial layers^2,9,31–33^. Moreover, radially situated clonally related excitatory neurons preferentially develop specific synapses with each other than with nearby non-clonally related excitatory neurons and share similar physiological properties^34–38^. While TBR2^+^ IPs play an essential role in expanding cortical neurogenesis, it remains unclear whether TBR2 regulates the spatial organization and synapse development of excitatory neurons in the cortex. In fact, how neurogenesis expansion is coordinated with synapse formation and functional organization of neurons is a fundamental question towards understanding cortical development and evolution.

In this study, we quantitatively examined the contribution of TBR2^+^ IPs to the neuronal and glial output of individual RGPs by performing mosaic analysis with double markers (MADM). We found that removal of TBR2 leads to a similar and significant loss of both deep and superficial layer excitatory neurons, but not glial cells, generated by individual RGPs. Moreover, in the absence of TBR2, clonally related excitatory neurons originating from individual RGPs become more laterally dispersed and their preferential synapse development is impaired. Mechanistically, we found that TBR2 binds to the genomic regulatory sequences upstream and downstream of *Protocadherin-19* (*Pcdh19*), a member of the *Cadherin* superfamily that has been implicated in female infantile-onset epilepsy and cognitive impairment and regulates its expression. Suppression of *Pcdh19* expression leads to defects in neuronal production and organization by individual RGPs similar to those observed following TBR2 removal. Furthermore, simultaneous PCDH19 expression rescues the defects in production, precise spatial organization and synaptic connectivity of cortical excitatory neurons caused by TBR2 loss. Together, these results reveal a critical molecular pathway involving TBR2 and PCDH19 in coordinating neurogenesis expansion and fine-scale circuit organization in the mammalian cortex.

## Results

### TBR2 removal reduces neuronal output by individual RGPs

To assess the precise contribution of TBR2^+^ IPs to cortical histogenesis, we took advantage of the *Tbr2* floxed mutant mice*, Tbr2*^*fl/fl*^^39^, and performed clonal analysis using MADM^40^. We introduced the *Emx1-CreER*^*T2*^ transgene^41^ into the MADM mice^9,42^ to specifically label RGPs in the dorsal telencephalon in a temporally controlled manner. In particular, Cre recombinase-driven inter-chromosomal recombination in the G_2_ phase of the dividing RGPs followed by X-segregation (G_2_-X) reconstitutes one of the two fluorescent markers, enhanced green fluorescent protein (EGFP, green) or tandem dimer Tomato (tdTomato, red), in each of the two daughter cells (Supplementary Fig. 1a). The permanent and distinct labeling of the two daughter cells and their respective progeny allows explicit analysis of the division pattern (symmetric vs. asymmetric) and potential (the number of neural progeny) of the originally labeled dividing RGPs.

We integrated the *Tbr2*^*fl/fl*^ allele with the *Emx1-CreER*^*T2*^*/MADM* system (Supplementary Fig. 1b). As shown previously^9^, we optimized the dose of TM to achieve a sparse labeling of individual dividing RGPs and corresponding clones in the cortex. We selectively focused on G_2_-X green/red fluorescent clones, as they reliably reflect the division pattern and neurogenic potential of individual dividing RGPs. To examine the contribution of TBR2^+^ IPs to cortical neurogenesis by individual RGPs, we administered TM to timed pregnant females at the following four embryonic stages: E10, E11, E12, and E13, performed cesarean section and rescue at ~E19, and collected the brain for analysis at postnatal day (P) 21 (Supplementary Fig. 1b). As expected, TM administration triggered reliable sparse labeling of individual green/red fluorescent clonal clusters as well as effective removal of TBR2 in the mutant embryonic cortex compared with the littermate control cortex (Supplementary Fig. 1c, d).

**Fig. 1:**
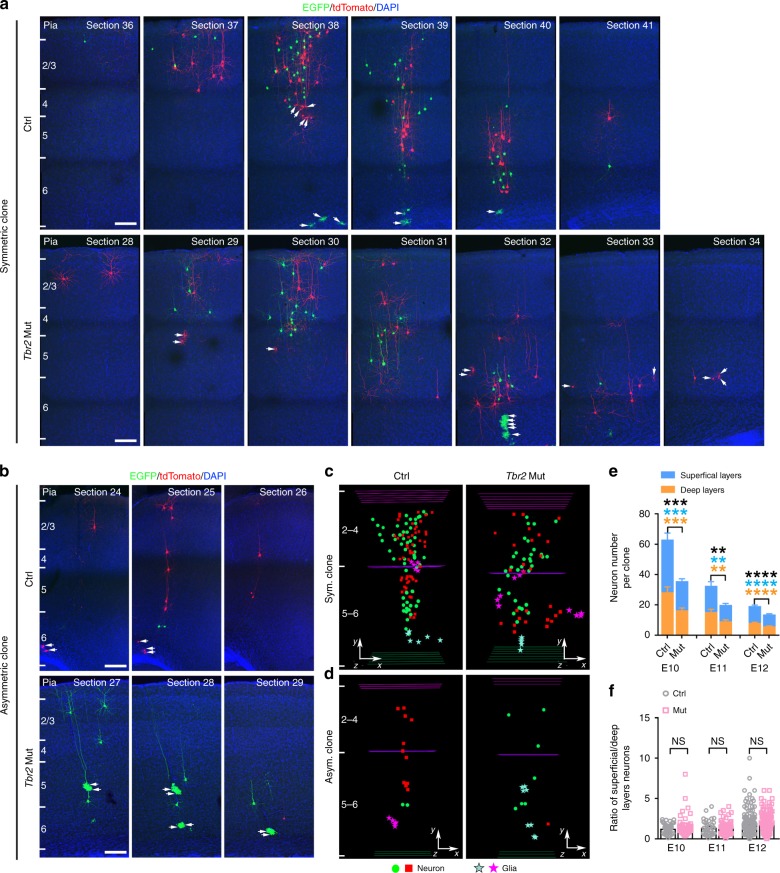
TBR2 removal causes a reduction in excitatory neuronal output of individual RGPs. **a**, **b** Confocal images of representative P21 control (Ctrl) and *Tbr2* mutant (Mut) symmetric (**a**) and asymmetric (**b**) MADM clones labeled by tamoxifen administration at E10 and E11, respectively. Consecutive sections were stained for EGFP (green) and tdTomato (red), and counter-stained with DAPI (blue). Arrows indicate glial cells. Scale bars: 100 μm. **c**, **d** 3D reconstruction images of the Ctrl and *Tbr2* Mut symmetric and asymmetric clones shown in (**a**) and (**b**). Different colored lines indicate the layer boundaries and different colored symbols represent the cell bodies of labeled neurons and glial cells. The *x*-/*y*-/*z*-axes indicate the spatial orientation of the clone with the *x*-axis parallel to the brain pial surface and *y*-axis perpendicular to the pial surface. Similar displays are used in subsequent 3D reconstruction images. **e** Quantification of the number of neurons in the Ctrl and *Tbr2* Mut symmetric and asymmetric clones (E10: Ctrl, *n* = 63; Mut, *n* = 78; E11: Ctrl, *n* = 35; Mut, *n* = 61; E12: Ctrl, *n* = 156; Mut, *n* = 154) labeled at different embryonic stages (E10-E12). Note the significant reduction in both deep (5–6, orange) and superficial (2–4, blue) layer neurons in the *Tbr2* Mut clone compared with the Ctrl clone. Asterisks indicate the statistical significance of the differences in the number of total (black), deep (orange), or superficial (blue) layer neurons in individual clones. Similar displays are used in subsequent figures. **f** Quantification of the ratio of superficial (2–4) versus deep (5–6) layer neuron numbers in the Ctrl and *Tbr2* Mut symmetric and asymmetric clones. Data are presented as mean ± SEM (**P < 0.01; ***P < 0.001; ****P < 0.0001; NS not significant; unpaired Student’s *t*-test)

To reveal all labeled cells in the cortex, we performed serial sectioning, immunohistochemistry, and three-dimensional (3D) reconstruction of individual brains (Supplementary Fig. 1b). RGPs divide either symmetrically to amplify themselves or asymmetrically to produce neurons or IPs while renewing. Consistent with this, we observed two major types of green/red fluorescent clonal clusters in the control (Ctrl) and *Tbr2* mutant (Mut) cortices (Fig. 1a, b). One type was the symmetric proliferative clone containing a large cohort of green or red fluorescent neuronal progeny spanning both deep (5–6) and superficial (2–4) layers, originating from the two daughter cells of symmetrically dividing RGPs that inherited reconstituted *EGFP* and *tdTomato*, respectively (Fig. 1a, c). The other type was the asymmetric neurogenic clone containing a minority population in one color typically situated in the deep layers and a majority population in the other color spanning both deep and superficial layers (Fig. 1b, d). In these clones, the majority population arises from a self-renewing RGP, whereas the minority population arises from a neuron or an IP. We found that, as the labeling (i.e., TM treatment) time proceeded from E10 to E13, the percentage of labeled clones that were symmetrically proliferative progressively decreased, while the percentage of labeled clones that were asymmetrically neurogenic concomitantly increased (Supplementary Fig. 2a). Notably, there was no obvious difference in the fraction of symmetrically proliferative versus asymmetrically neurogenic clones in the Ctrl or *Tbr2* Mut cortices at different embryonic stages (Supplementary Fig. 2a), indicating that removal of TBR2 does not affect the division mode of RGPs.

We next systematically compared the number of neurons in individual Ctrl and *Tbr2* Mut clones labeled at different embryonic stages. We found that the average number of neurons in the *Tbr2* Mut clone was substantially smaller than in the Ctrl clone across different labeling times (Fig. 1e) and clonal types (Supplementary Fig. 2b, c). Moreover, the reduction was significant and comparable for deep (5–6) and superficial (2–4) layer neurons (Fig. 1e and Supplementary Fig. 2b, c). As a result, the relative ratio of superficial versus deep layer neurons in individual clones was similar between the Ctrl and *Tbr2* Mut clones (Fig. 1f and Supplementary Fig. 2d, e). Together, these results demonstrate that at the single RGP level TBR2^+^ IPs contribute significantly and similarly to the production of both deep and superficial layer neurons in the cortex.

Consistent with this, we observed significant reductions in both deep and superficial layer neurons in the *Tbr2* conditional knockout (cKO) cortices using *Emx1-Cre*^43^ (Supplementary Fig. 3). We also observed a significant reduction in the number of mitotic cells in the SVZ of the *Tbr2* cKO cortex at the embryonic stage (Supplementary Fig. 4a, b), indicating a loss of IPs in the absence of TBR2, as suggested in the previous studies^25,27^. There was no obvious difference in cell death between the control and *Tbr2* cKO cortices at the embryonic and postnatal stages (Supplementary Fig. 4c).

RGPs also produce glial cells. We often observed green or red fluorescent glial cells in the labeled clonal clusters, including both astrocytes and oligodendrocytes (Fig. 1a, b, arrows and Supplementary Fig. 5a, b). To test whether TBR2 regulates glial cell generation by individual RGPs, we examined the numbers of glial cells in individual Ctrl and *Tbr2* Mut clones and found that they were comparable (Supplementary Fig. 5c–f). These results suggest that TBR2 removal does not affect glial cell output of individual RGPs in the developing cortex.

### TBR2 removal causes excitatory neuron clone dispersion

**Fig. 2:**
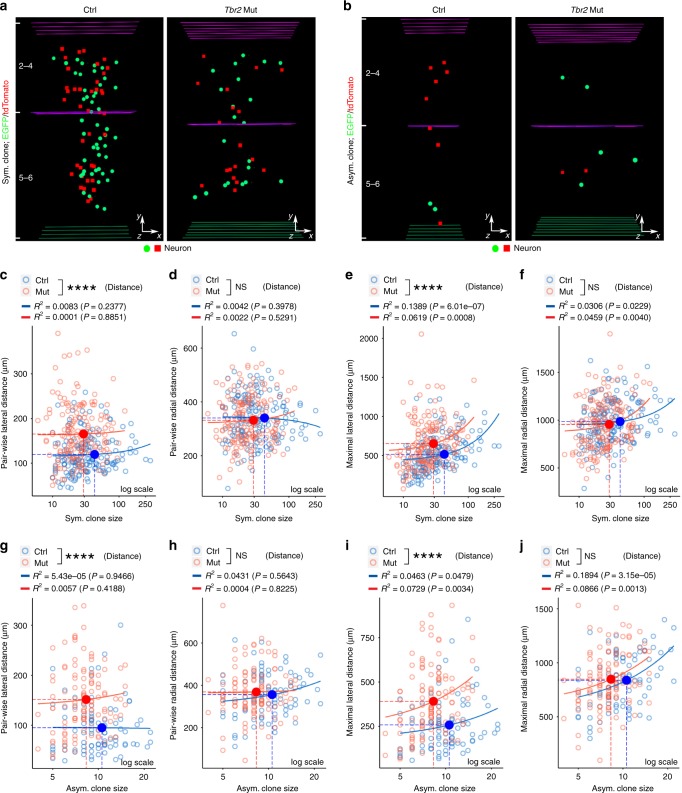
TBR2 removal causes a lateral dispersion of clonally related excitatory neurons. **a**, **b** 3D reconstruction images of representative P21 Ctrl (left) and *Tbr2* Mut (right) symmetric (**a**) and asymmetric (**b**) MADM clones. **c**–**f** Quantification of the pair-wise (**c**, **d**) and maximal (**e**, **f**) lateral and radial distances between neurons in symmetric Ctrl (blue) and *Tbr2* Mut (red) clones (Ctrl, *n* = 169; Mut, *n* = 177). The *X*-axis is in log scale. Each open circle represents an individual clone and the filled circles and broken lines indicate the means of distances and clone sizes of each group. The solid lines represent linear regression between clone size and distances of each group. Similar displays are used in subsequent figures. **g**–**j**, Quantification of the pair-wise (**g**, **h**) and maximal (**i**, **j**) lateral and radial distances between neurons in asymmetric Ctrl and *Tbr2* Mut clones (Ctrl, *n* = 85; Mut, *n* = 116). Data are presented as mean ± SEM (****P < 0.0001; NS not significant; unpaired Student’s *t*-test)

Clonally related excitatory neurons arising from the same dividing RGPs migrate radially and form discrete ontogenetic radial clusters in the cortex^2,9,31–33^. Interestingly, we found that the spatial distribution of the *Tbr2* Mut clone was clearly altered compared with the Ctrl clone (Fig. 2). While forming spatially isolated radial clusters occupying deep and superficial layers, clonally related excitatory neurons in individual *Tbr2* Mut clones became more laterally dispersed compared with those in the Ctrl clone (Fig. 2a, b).

To quantitatively assess this, we analyzed the pair-wise and maximal lateral and radial distances between clonally related neurons in individual Ctrl and *Tbr2* Mut clones. Compared with the control, the pair-wise and maximal lateral distances between clonally related neurons in the *Tbr2* Mut clone were significantly increased (Fig. 2c, e, g, i), whereas the pair-wise and maximal radial distance remained comparable (Fig. 2d, f, h, j), for both symmetric proliferative and asymmetric neurogenic clones. Notably, the increases in the pair-wise and maximal lateral distances were evident for clones with different numbers of neurons (Supplementary Fig. 6), indicating that the lateral dispersion of the *Tbr2* Mut clone is not due to the decrease in clonal size. There was no obvious correlation between the pair-wise lateral distance and the clone size. The increase in lateral dispersion was evident for both deep and superficial layer neurons (Supplementary Fig. 7). Notably, we observed an increase in the number of neurites in post-mitotic neurons in the *Tbr2* Mut clone compared with those in the control clone (Supplementary Fig. 8), which has been associated with abnormal tangential migration and spread in previous studies^44,45^. Together, these results suggest that TBR2 regulates the precise spatial distribution of clonally related excitatory neurons in the cortex.

### TBR2 removal disrupts preferential synaptic connectivity

**Fig. 3:**
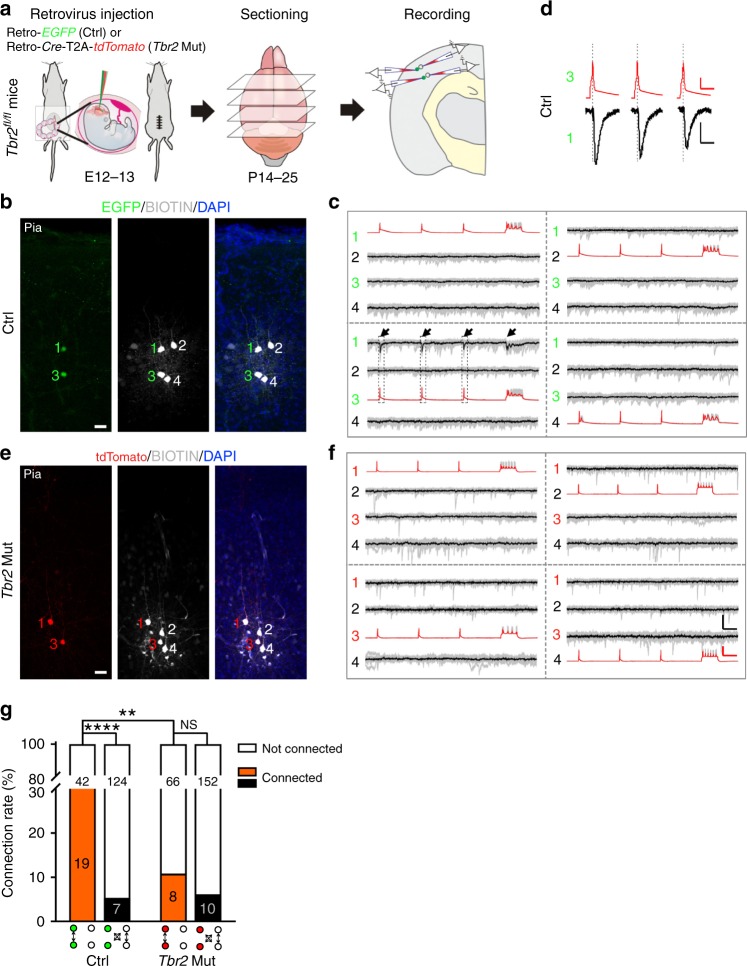
TBR2 removal impairs preferential chemical synaptic connectivity. **a** Schematic diagram of in utero intraventricular injection of retrovirus expressing EGFP (green, Ctrl) or Cre/tdTomato (red, *Tbr2* Mut) into the *Tbr2*^*fl/fl*^ mice at E12-13. Acute brain slices were prepared at P14–25 for quadruple whole-cell patch-clamp recording. **b**, **e** Representative confocal images of two radially situated EGFP-expressing sister excitatory neurons (1 and 3, green, Ctrl, **b**) or Cre/tdTomato-expressing sister excitatory neurons (1 and 3, red, *Tbr2* Mut, **e**) and two adjacent unlabeled non-sister excitatory neurons (2 and 4). Scale bars: 50 μm. **c**, **f** Example traces of four excitatory neurons recorded in (**b**) and (**e**), respectively. Brief and long duration depolarizing currents were injected into one of the four neurons to elicit action potentials (presynaptic, red) and postsynaptic responses were monitored in the other three neurons. Individual traces are shown in gray and average traces are shown in black. Arrows indicate the reliable postsynaptic responses in control sister excitatory neuron 1 elicited by the presynaptic action potentials in control sister excitatory neuron 3. Scale bars: 100 mV (red), 20 pA (black), and 200 ms. **d** Zoom-in traces of the presynaptic action potentials and postsynaptic responses between EGFP-expressing control sister excitatory neurons 1 and 3 in (**c**). Scale bars: 25 mV (red), 10 pA (black), and 25 ms. **g** Summary of the frequency of chemical synaptic connections between the control (green) or *Tbr2* mutant (red) sister excitatory neurons and nearby non-sister excitatory neurons. The numbers of recorded pairs are shown in the bar graph. A similar display is used in subsequent figures (**P < 0.01; ****P *<* 0.0001; NS not significant; chi-square test)

Previous studies showed that clonally related excitatory neurons arising from the same neurogenic RGPs (i.e., sister excitatory neurons) not only form spatially isolated radial clusters^2,9,31–33^ but also preferentially develop chemical synapses with each other over nearby non-clonally related excitatory neurons in the cortex^34,35,38^. We next asked whether TBR2 regulates the preferential synaptic connectivity between sister excitatory neurons originating from the same neurogenic RGPs. To address this, we performed in utero intraventricular injection of low-titer retroviruses expressing EGFP or Cre/tdTomato into the *Tbr2*^*fl/fl*^ mouse embryos at E12-13 (Fig. 3a), when cortical RGPs have largely transitioned from the proliferation phase to the neurogenesis phase to produce excitatory neurons^9^. In these experiments, the sparse infection of individual dividing RGPs at the VZ surface by EGFP or Cre/tdTomato-expressing retroviruses resulted in the labeling of individual excitatory neuron clones in the cortex. The co-existence of both control/EGFP and *Tbr2* Mut/tdTomato clones in the same brain allowed for an effective and direct comparison of their synaptic connectivity in the same experiments. Notably, the control and *Tbr2* Mut clones labeled by low-titer retrovirus infection and MADM at E12-13 were similar in size and laminar distribution (Supplementary Fig. 9 and 10), indicating that low-titer retroviral infection is a consistent and reliable method for labeling individual excitatory neuron clones in the cortex originating from single neurogenic RGPs.

To assess preferential synaptic connectivity between clonally related excitatory neurons, we prepared acute cortical brain slices at P14–25, when excitatory chemical synapses are largely formed^34,35^, and performed quadruple whole-cell patch-clamp recordings onto EGFP (Ctrl) or Cre/tdTomato (*Tbr2* Mut)-expressing clonally related excitatory neurons and nearby unlabeled excitatory neurons as non-clonally related control excitatory neurons in the somatosensory and visual cortices across deep and superficial layers (Fig. 3b, e). We first examined the biophysical properties of the recorded neurons and found that the resting membrane potential, the threshold for firing action potential, and the maximal firing rate of the Ctrl and *Tbr2* Mut neurons were largely similar (Supplementary Fig. 11a–d), indicating that TBR2 removal does not affect membrane properties and maturation of cortical excitatory neurons.

To test whether clonally related excitatory neurons preferentially develop chemical synapses with each other, we simultaneously recorded from two sparsely labeled EGFP (Ctrl, Fig. 3b) or Cre/tdTomato (*Tbr2* Mut, Fig. 3e)-expressing excitatory neurons in a spatially isolated radial/vertical cluster (1 and 3) and two nearby unlabeled, non-clonally related control excitatory neurons (2 and 4). The control excitatory neurons were selected based on their morphological characteristics, including a pyramid-shaped cell body with a major apical dendrite. Once all recordings were established, the excitatory neuron identity of recorded cells was further confirmed by their morphological and electrophysiological properties. Trains of brief and extended suprathreshold depolarizing currents were injected sequentially into one of the four neurons to trigger action potentials, and the current changes were monitored in all the other three neurons to probe chemical synaptic connectivity (Fig. 3c, f). In the control clone example shown here (Fig. 3c, d), action potentials generated in EGFP-expressing excitatory neuron 3 reliably elicited postsynaptic responses in its clonally related EGFP-expressing excitatory neuron 1, indicating the existence of a chemical synaptic connection between them. This connection was unidirectional, as action potentials generated in EGFP-expressing excitatory neuron 1 failed to elicit detectable postsynaptic responses in its clonally related EGFP-expressing excitatory neuron 3. No obvious postsynaptic response was observed when the non-EGFP-expressing excitatory neuron 2 or 4 was depolarized to fire action potentials. Together, these results suggest that clonally related excitatory neurons 1 and 3 in the same radial cluster are selectively synaptically connected.

We analyzed a total of 61 quadruple recordings of clonally related, radially situated EGFP-expressing Ctrl excitatory neuron pairs, as well as nearby unlabeled non-clonally related control excitatory neurons (Fig. 3g, left). Of the clonally related EGFP-expressing neuron pairs, ~31.1% (19 out of 61) were synaptically connected. In contrast, only ~5.3% (7 out of 131) of the control non-clonally related excitatory neuron pairs were connected. Together, these results suggest that clonally related excitatory neurons originating from the same neurogenic RGPs preferentially develop chemical synapses with each other over nearby non-clonally related excitatory neurons in the cortex, as shown previously^34^.

In comparison, we analyzed a total of 74 quadruple recordings of clonally related, radially situated Cre/tdTomato-expressing *Tbr2* Mut excitatory neuron pairs and nearby unlabeled non-clonally related control excitatory neurons (Fig. 3g, right). Of the clonally related Cre/tdTomato-expressing *Tbr2* Mut neuron pairs, only ~10.8% (8 out of 74) were synaptically connected, comparable to the connection rate of the non-clonally related excitatory neuron pairs (~6.2%, 10 out of 162), indicating that clonally related *Tbr2* Mut excitatory neurons do not preferentially develop chemical synapses with each other. Notably, the connection rate of clonally related *Tbr2* Mut excitatory neuron pairs (~10.8%) was significantly lower than that of clonally related Ctrl excitatory neuron pairs (~31.1%) (Fig. 3g), suggesting that TBR2 removal disrupts preferential synapse development between clonally related excitatory neurons. The inter-soma distances between clonally related and unrelated Ctrl and *Tbr2* Mut excitatory neuron pairs were largely similar (EGFP/EGFP: 199.8 ± 22.0 µm versus tdTomato/tdTomato: 187.3 ± 12.3 µm; *P* *=* 0.95; tdTomato/non-tdTomato or non-tdTomato/non-tdTomato: 186.0 ± 8.4 µm versus tdTomato/tdTomato: 187.3 ± 12.3 µm; *P* = 0.99; Data are presented as Mean ± SEM; Student’s *t*-test). We did not observe any obvious differences in dendritic morphology between the Ctrl and *Tbr2* Mut excitatory neurons (Supplementary Fig. 11e–g). The recorded neurons were similarly distributed across deep and superficial layers (Supplementary Fig. 11h). Together, these results strongly suggest that TBR2 regulates preferential chemical synapse formation between clonally related excitatory neurons in the cortex.

### TBR2 regulates PCDH19 expression

**Fig. 4:**
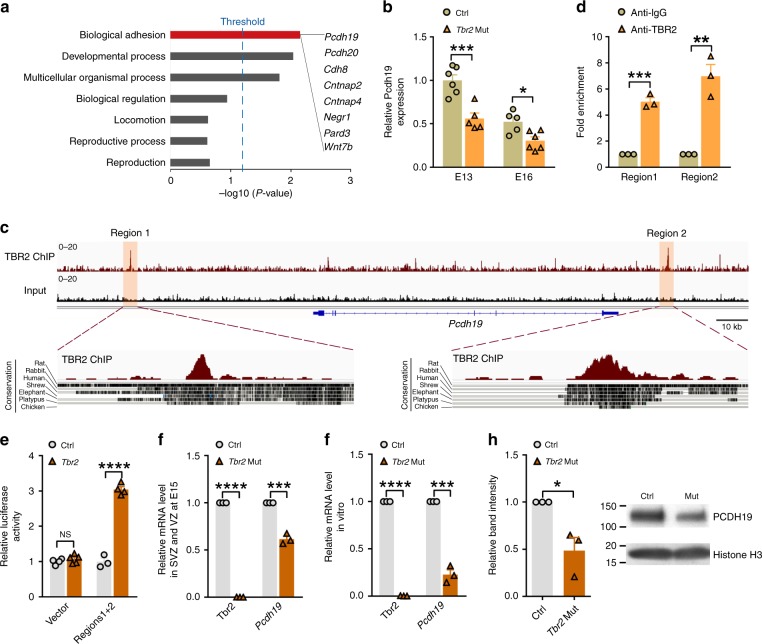
TBR2 regulates *Pcdh19* expression by two evolutionarily conserved binding sites. **a** Overrepresentation analysis of differentially expressed genes in the *Tbr2* Mut IPs compared with the control IPs based on microarray analysis. Threshold: Benjamini adjusted *p* value < 0.05. **b** Reduced *Pcdh19* expression in the *Tbr2* Mut IPs compared with the control at E13 and E16 based on microarray analysis (*n* = 5–6) (*P < 0.05, ***P < 0.001; two-way ANOVA, Benjamini Hochberg FDR used for *P* value adjustment). **c** Two evolutionarily conserved TBR2 binding sequences located upstream and downstream of *Pcdh19* based on ChIP-seq analysis in the E14 mouse cortex. The binding sequences 1 and 2 are highlighted (regions 1 and 2) and evolutionarily conserved regions are shown at the bottom. **d** The enrichment of TBR2-bound sequences 1 and 2 upstream and downstream of *Pcdh19* determined by ChIP-qPCR in the E14 mouse cortex (*n* = 3). **e** Increase in the relative luciferase activity driven by the TBR2 binding sequences 1 and 2 upstream and downstream of *Pcdh19* in the presence of TBR2 (*n* = 4). **f** Decrease in *Pcdh19* mRNA in the *Tbr2* mutant cortex at E15 compared with the control determined by RT-qPCR (*n* = 3). **g** Decrease in *Pcdh19* mRNA in the *Tbr2* mutant cortical neural progenitors cultured in vitro compared with the control determined by RT-qPCR (*n* = 3). **h** Decrease in PCDH19 protein expression in the *Tbr2* mutant cortical neural progenitors cultured in vitro compared with the control determined by Western blot (*n* = 3). Data are presented as mean ± SEM. (*P < 0.05; **P < 0.01; ***P < 0.001; ****P < 0.0001; NS not significant; unpaired Student’s *t*-test)

Having found that TBR2 regulates the production and precise spatial and synaptic organization of clonally related excitatory neurons in the cortex, we next investigated the underlying molecular mechanisms. To achieve this, we used the *Tbr2-EGFP* reporter mice^46^ as well as *Tbr2*^*fl/fl*^ mice and isolated EGFP-labeled IPs from the control and *Tbr2* mutant cortices at E13 and E16. We then extracted RNA and performed microarray analysis to assess genes that were differentially expressed in the control and *Tbr2* mutant IPs. Interestingly, analysis of the set of differentially expressed genes for significant enrichment of biological processes yielded the term ‘biological adhesion’ (Fig. 4a and Supplementary Data 1), of which *Pcdh19* was one of the top genes that showed a strongly reduced expression in the *Tbr2* mutant IPs compared with the control (Fig. 4a, b and Supplementary Data 2). *Pcdh19*, encoding a member of the Cadherin superfamily^47^, affecting the columnar cellular organization in the zebrafish optic tectum and the spatial distribution of neurons in the mouse cortex^48–50^. These observations raise the intriguing possibility that TBR2 regulates the expression of *Pcdh19* in the developing cortex and consequently controls cortical neurogenesis and precise spatial and functional neuronal organization. To test this, we took advantage of the recently published chromatin immunoprecipitation sequencing (ChIP-seq) dataset of TBR2 in E14 mouse cortex^51^ and analyzed its genomic binding profile. Interestingly, we identified two prominent TBR2 binding peaks located in the proximity of the *Pcdh19* gene, one located upstream (Region 1) and the other downstream (Region 2) of its genomic locus (Fig. 4c). Notably, both binding regions are evolutionarily conserved, indicating their potential importance in regulating *Pcdh19* expression by TBR2.

To determine whether TBR2 does indeed bind to these two regions, we carried out ChIP-quantitative PCR (ChIP-qPCR) analysis using a TBR2 antibody in E14 mouse cortex and observed obvious enrichment of the two binding sequences near *Pcdh19* gene (Fig. 4d), suggesting that TBR2 binds to these two regions. To test whether TBR2 binds directly to these two regions and consequently regulates *Pcdh19* expression, we cloned these two binding regions into a luciferase reporter vector and examined TBR2 regulatory activity in cultured mammalian cells. We found that TBR2 strongly enhanced luciferase activity (Fig. 4e). Together, these results suggest that TBR2 binds to the genomic sequences located upstream and downstream of *Pcdh19* and promotes its expression in the developing cortex.

Consistent with this, we found that *Pcdh19* mRNA was significantly decreased in the *Tbr2* mutant embryonic cortex compared with the control, based on real-time quantitative reverse transcription-PCR (qRT-PCR) analysis (Fig. 4f). To further confirm the effect of TBR2 removal on PCDH19 expression, we isolated neural progenitor cells from E12 mouse cortex and prepared cultures in vitro. We found that *Pcdh19* mRNA and PCDH19 protein in the *Tbr2* cKO progenitor cell culture were significantly reduced compared with the control (Fig. 4g, h and Supplementary Fig. 12a, b). We also observed a reduction in PCDH19 expression in the SVZ of the *Tbr2* cKO cortex compared with the control at E15 (Supplementary Fig. 12c, d and Supplementary Fig. 13a, b). Collectively, these results strongly suggest that TBR2 regulates the expression of PCDH19 in the developing cortex.

### PCDH19 regulates neurogenesis and neuronal organization

We next examined the role of PCDH19 in controlling cortical neurogenesis and neuronal organization. We engineered four short hairpin RNAs (shRNAs) against mouse *Pcdh19* and evaluated their efficacy in suppressing PCDH19 expression. Of the four *Pcdh19* shRNAs, shRNA-4 exhibited the strongest suppression efficiency (Supplementary Fig. 14a). We then generated retroviruses expressing control or *Pcdh19* shRNA-4 together with EGFP, performed in utero intraventricular injection of low-titer retroviruses at E11, and examined the number and spatial distribution of individual excitatory neuron clones in the cortex at P21.

**Fig. 5:**
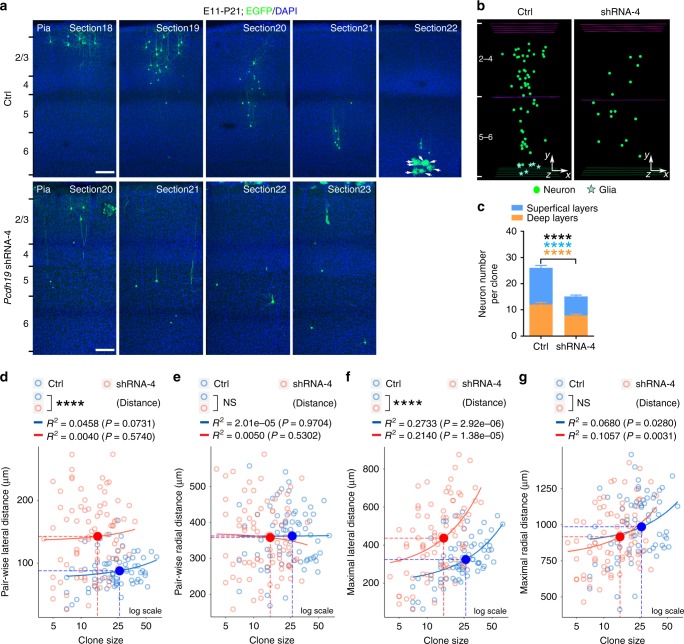
PCDH19 knockdown causes neuronal and lateral dispersion defects. **a** Representative confocal images of P21 excitatory neuron clones expressing EGFP and control shRNA (top) or *Pcdh19* shRNA-4 (bottom). Arrows indicate glial cells. Scale bars: 100 μm. **b** 3D reconstruction images of the control and *Pcdh19* shRNA-4 clones shown in (**a**). **c** Quantification of the number of neurons in individual clones expressing control (*n* = 71) or *Pcdh19* shRNA-4 (*n* = 81). **d**, **e** Quantification of the pair-wise lateral and radial distances between neurons in clones expressing control (*n* = 71) or *Pcdh19* shRNA-4 (*n* = 81). **f**, **g** Quantification of the maximal lateral and radial distances between neurons in clones expressing control (*n* = 71) or *Pcdh19* shRNA-4 (*n* = 81). Data are presented as mean ± SEM. (****P < 0.0001; NS not significant; unpaired *t*-test)

Compared with the control excitatory neuron clone, the excitatory neuron clone expressing *Pcdh19* shRNA-4 exhibited clear differences in both the number and spatial distribution of clonally related neurons (Fig. 5a, b). The number of neurons in the *Pcdh19* shRNA-4-expressing clone was significantly smaller than that in the control clone (Fig. 5c). This decrease was obvious for both superficial and deep layer neurons. Moreover, clonally related neurons in the *Pcdh19* shRNA-4-expressing clone were more laterally dispersed than those in the control clone, as evidenced by the significant increases in the pair-wise and maximal lateral, but not radial, distances (Fig. 5d–g). To confirm that the observed effects were attributable specifically to *Pcdh19* knockdown, we mutated the *Pcdh19* shRNA-4 sequence (shRNA-4^S^), which failed to suppress *Pcdh19* expression (Supplementary Fig. 14b, c). No obvious defects in neuronal production (Supplementary Fig. 14d–f) or spatial organization (Supplementary Fig. 14g–j) were observed in the *Pcdh19* shRNA-4^S^-expressing clones, suggesting that the defects observed in the *Pcdh19* shRNA-4 expressing clones are due to a selective suppression of PCDH19 expression. Notably, suppression of PCDH19 expression also led to an increase in the number of neurites in new-born neurons in the SVZ and IZ at the embryonic stage (Supplementary Fig. 15a, b), but not in mature neurons at P21 (Supplementary Fig. 15c–e). Together, these results suggest that suppression of PCDH19 expression causes a significant reduction in neuronal output by individual RGPs and a lateral dispersion of clonally related neurons in the cortex, mimicking the effects of TBR2 removal. Consistent with this, we observed a significant reduction in the number of TBR2^+^ IPs in the developing cortex expressing *Pcdh19* shRNA (Supplementary Fig. 16), indicating that suppression of PCDH19 expression results in a loss of IPs.

To further examine the role of PCDH19 in regulating cortical neurogenesis and neuronal organization, we engineered retroviruses to overexpress PCDH19 together with tdTomato as two separate proteins (Supplementary Fig. 17). We found that compared with the control, PCDH19 overexpression led to a significant increase in the number of excitatory neurons in individual clones labeled at E10-11 (Supplementary Fig. 17a–c). Moreover, clonally related neurons became more laterally clustered, as reflected in the significant decrease in the pair-wise and maximal lateral distances (Supplementary Fig. 17d–g). Interestingly, we also observed a significant increase in the synaptic connectivity between clonally related excitatory neurons overexpressing PCDH19 (Supplementary Fig. 18). Together, these results suggest that PCDH19 plays critical roles in regulating cortical neurogenesis and neuronal spatial and synaptic organization.

### PCDH19 rescues neurogenesis and spatial organization defects

**Fig. 6:**
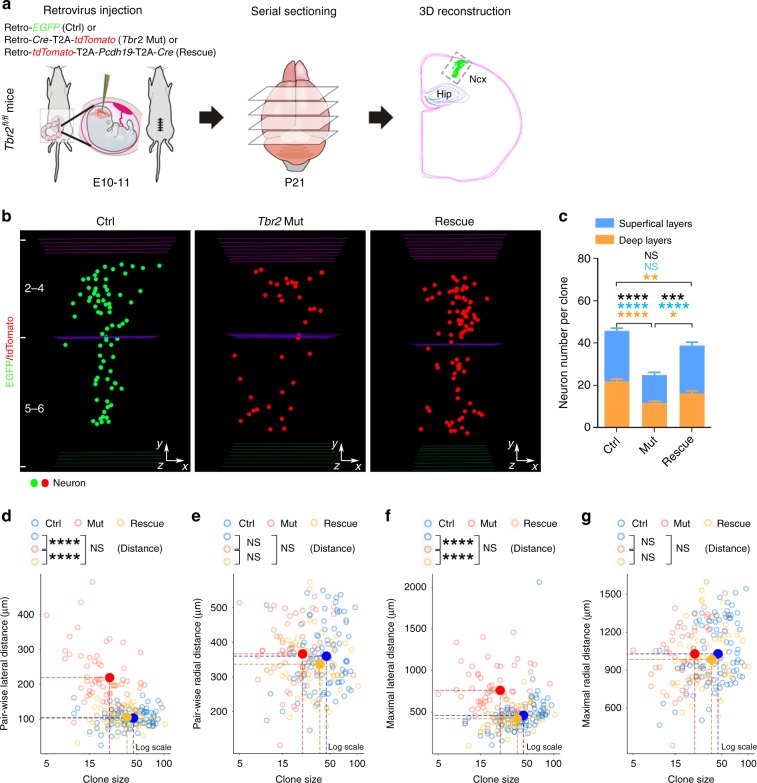
PCDH19 rescues the defects in clonal neuronal production and spatial distribution. **a** Schematic diagram of in utero intraventricular injection of low-titer retroviruses expressing EGFP (green, Ctrl), Cre/tdTomato (red, *Tbr2* Mut), or tdTomato/PCDH19/Cre (red, Rescue) into the *Tbr2*^*fl/fl*^ mice at E11. Brains were collected at P21 and subjected to serial sectioning and 3D reconstruction to recover individual clones in the cortex. **b** Representative 3D reconstruction images of control (left, green), *Tbr2* Mut (middle, red), and Rescue (right, red) clones. **c**, Quantification of the number of neurons in the control (*n* = 74), *Tbr2* Mut (*n* = 62), and Rescue (*n* = 53) clones. **d**–**g** Quantification of the pair-wise (**d** and **e**) and maximal (**f** and **g**) lateral and radial distances between neurons in control (blue, *n* = 74), *Tbr2* Mut (red, *n* = 62), and Rescue (yellow, *n* = 53) clones. Data are presented as mean ± SEM. (*P < 0.05; **P < 0.01; ***P < 0.001; ****P < 0.0001; NS not significant; one-way ANOVA followed by Tukey’s test)

We next examined the functional interaction between PCDH19 and TBR2. In particular, we tested whether PCDH19 expression could rescue the defects caused by TBR2 removal. To achieve this, we engineered retroviruses simultaneously expressing tdTomato/PCDH19/Cre as separate proteins and performed in utero intraventricular injection into the *Tbr2*^*fl/fl*^ mouse embryo at E10-11 (Fig. 6a). These retroviruses label excitatory neuron clones in the cortex with tdTomato while selectively deleting *Tbr2* and simultaneously expressing PCDH19 (Rescue). In these experiments, we also used retroviruses expressing tdTomato/Cre to label the *Tbr2* Mut clone and retroviruses expressing EGFP alone to label the wild type Ctrl clone. Brains were collected at P21 and individual excitatory neuron clones were recovered for systematic analysis.

Consistent with our MADM results (Fig. 1), the Cre/tdTomato-expressing *Tbr2* Mut clone contained fewer excitatory neurons that were more laterally dispersed than the EGFP-expressing wild type Ctrl clone (Fig. 6b–g). Remarkably, we observed more neurons in the tdTomato/PCDH19/Cre-expressing (Rescue) clone than in the tdTomato/Cre-expressing (*Tbr2* Mut) clone (Fig. 6b, c). Moreover, clonally related neurons in the Rescue clone became less laterally dispersed than the *Tbr2* Mut clone, similar to the Ctrl wild type clone (Fig. 6b, d–g). Together, these results suggest that the defects in clone size and spatial distribution caused by TBR2 removal are largely rescued by simultaneous expression of PCDH19. The similar clonal phenotype between TBR2 removal and PCDH19 knockdown and the effective rescue of TBR2 removal phenotype by PCDH19 expression strongly suggest that PCDH19 functionally interacts with TBR2 in controlling the production and precise spatial organization of excitatory neurons in the cortex.

### PCDH19 restores preferential synaptic connectivity defect

**Fig. 7:**
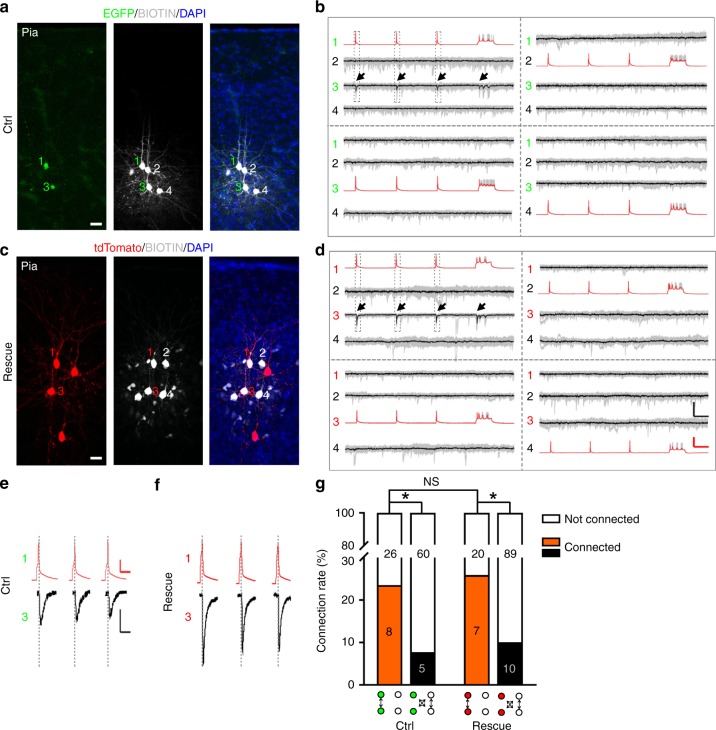
PCDH19 rescues the preferential chemical synaptic defect. **a**, **c** Representative confocal images of two radially situated sister excitatory neurons in a control clone expressing EGFP (1 and 3, green, **a**) or in a rescue clone expressing tdTomato/PCDH19/Cre (1 and 3, red, **c**) and two adjacent non-EGFP or non-tdTomato-expressing non-sister excitatory neurons (2 and 4) in the *Tbr2*^*fl/fl*^ mice. Scale bars: 50 μm. **b**, **d** Example traces of four excitatory neurons recorded in (**a**) and (**c**), respectively. Brief and long duration depolarizing currents were injected into one of the four neurons to elicit action potentials (presynaptic, red) and postsynaptic responses were monitored in the other three neurons. Individual traces are shown in gray and average traces are shown in black. Arrows indicate the reliable postsynaptic responses in control or rescue sister excitatory neuron 3 elicited by the presynaptic action potentials in control or rescue sister excitatory neuron 1. Scale bars: 100 mV (red), 20 pA (black), and 200 ms. **e**, **f** Zoom-in traces of the presynaptic action potentials and postsynaptic responses between EGFP-expressing control or tdTomato-expressing rescue sister excitatory neurons 1 and 3 in (**b**) and (**d**), respectively. Scale bars: 25 mV (red), 10 pA (black), and 25 ms. **g** Summary of the frequency of chemical synaptic connections between sister excitatory neurons and their nearby non-sister excitatory neurons in control and rescue clones. The numbers of the recorded pairs are shown in the bar graph (*P < 0.05; NS not significant; chi-square test)

Notably, as shown previously ^52^, PCDH19 was expressed in the cortex at the postnatal stage (Supplementary Fig. 12e and Supplementary Fig. 13c), when excitatory synapses actively form. In addition, we observed a clear reduction in PCDH19 expression in the postnatal *Tbr2* cKO cortex compared with the control (Supplementary Fig. 12e, f and Supplementary Fig. 13c, d). We next examined whether PCDH19 expression restores the preferential synaptic connectivity between clonally related excitatory neurons in the cortex lacking TBR2 by performing in utero injection of low-titer retroviruses expressing EGFP alone (Ctrl) or tdTomato/PCDH19/Cre (Rescue) into the *Tbr2*^*fl/fl*^ mouse embryo at E12-E13. After preparing acute cortical slices at P14–25, we carried out quadruple whole-cell recordings to examine the chemical synaptic connectivity between clonally related excitatory neurons and nearby non-clonally related excitatory neurons (Fig. 7a, c).

As shown earlier (Fig. 3), EGFP-expressing control clonally related excitatory neurons displayed a strong propensity for developing chemical synapses with each other over nearby non-EGFP-expressing non-clonally related excitatory neurons in a similar spatial configuration (clonally related: ~23.5%, 8 out 34 pairs; non-clonally related, ~5.9%, 5 out of 65 pairs) (Fig. 7b, e, g left), suggesting preferential synaptic connectivity between clonally related excitatory neurons in the cortex. Notably, tdTomato/PCDH19/Cre-expressing clonally related excitatory neurons expressing PCDH19 with simultaneous *Tbr2* deletion exhibited a similar propensity for developing chemical synapses with each other over nearby non-clonally related excitatory neurons (clonally related: ~25.9%, 7 out of 27 pairs; non-clonally related: ~4.9%, 10 out of 99) (Fig. 7d, f, g right). Together, these results suggest that PCDH19 expression restores the preferential synaptic connectivity between clonally related excitatory neurons disrupted by TBR2 removal.

## Discussion

Proper production and precise structural and functional organization of neurons are two fundamental aspects of mammalian cortical development and evolution. In this study, we employed MADM and performed an in-depth quantitative clonal analysis of the neural output of individual RGPs in the absence of TBR2. We found that removal of TBR2 leads to a substantial (~30–40%) reduction in the neuronal, but not glial, output of individual RGPs. Moreover, the reduction is significant and similar for both deep and superficial layer neurons. These results provide clear quantitative clonal evidence for TBR2^+^ IPs contributing to the generation of neurons across the deep and superficial layers, as suggested by previous studies^27–30^.

A prior lineage tracing study using the *Tbr2-Cre* knock-in mouse line showed that TBR2^+^ IPs contribute ~70–80% of cortical excitatory neurons in both deep and superficial layers^29^. Should TBR2 removal lead to a switch from indirect neurogenesis (~2 neurons) to direct neurogenesis (1 neuron), one would expect a ~30–40% reduction of excitatory neurons in the cortex, as our clonal analysis data demonstrated. These results are consistent with a recent embryonic MADM study showing that the average neuronal output of individual TBR2^+^ IPs is ~2.3 excitatory neurons^53^. Notably, a substantial population of dividing cells remains in the SVZ of the *Tbr2* mutant cortex, indicating the possible existence of non-TBR2^+^ IPs. It is unclear whether this is due to a compensatory regulation upon TBR2 removal. Nonetheless, at least ~70–80% of cortical excitatory neurons go through a stage of TBR2 expression, raising the possibility that TBR2 may regulate neuronal differentiation and organization in addition to supporting indirect neurogenesis via IPs.

Interestingly, we found that individual cortical excitatory neuron clones lacking TBR2 are not only smaller, but also more laterally dispersed. This lateral dispersion of clonally related neurons is not simply due to a decrease in neuronal number, as the dispersion is similar and evident for clones of different sizes. Notably, newborn neurons in the *Tbr2* mutant clone grow more neurites than the control, which likely contributes to abnormal migration and distribution of the clone, as suggested by previous studies^44,45^. Moreover, the preferential chemical synapse formation between clonally related neurons is also impaired. These results suggest that TBR2 not only regulates the number, but also controls the fine-scale spatial distribution and synaptic connectivity of excitatory neurons in the cortex. Notably, the lateral dispersion of the *Tbr2* mutant clone can be observed at P5–7, prior to the abundant formation and detection of preferential chemical synapses between clonally related excitatory neurons^34^. Given that neuronal migration and positioning affect preferential electrical synapse formation^38^, which regulates subsequent chemical synapse development^35^, it is possible that the two aspects of defects caused by TBR2 removal are related. Together, our data suggest that neuronal production and fine-scale structural and functional neuronal organization are highly coordinated in the mammalian cortex. Such coordination would be essential to cortical expansion during evolution. We did not observe any obvious change in glial cell output by individual RGPs in the absence of TBR2. This is consistent with the lineage tracing analysis using *Tbr2-Cre* which showed that TBR2^+^ IPs do not produce astrocytic cells in the neocortex^29^.

TBR2 is a transcription factor originally found to be required for mesoderm differentiation^54^. While it has been well-established to be a marker of IPs in the developing cortex, the molecular mechanisms related to its function in regulating cortical neurogenesis and development remain largely undefined. In human embryonic stem cells (hESCs), down-regulation of the pluripotency factors such as SOX2 and OCT4 induces the expression of TBR2, which in turn further represses the expression of pluripotency markers and drives differentiation^55^. In this context, TBR2 is found to be required for early embryonic expression of a number of genes, including *PDGFR*α and *Pcdh19*. Interestingly, our analysis of differential gene expression between wild type and TBR2-lacking IPs in the embryonic mouse cortex also identified *Pcdh19* as a primary TBR2 regulated gene. Based on the previously published ChIP-seq data in the embryonic mouse cortex^51^, we identified two TBR2 binding sequences located upstream and downstream of *Pcdh19* that were capable of mediating TBR2-dependent gene expression. Consistent with the notion that TBR2 regulates the expression of PCDH19 in the developing mouse cortex, TBR2 removal leads to a decrease in *Pcdh19* transcript and PCDH19 expression in cortical progenitors in culture and in vivo. Collectively, these results suggest that *Pcdh19* is a bone fide downstream target of TBR2. Remarkably, we found that *Pcdh19* knockdown results in a similar phenotype to *Tbr2* deletion. Compared with the control clone, the *Pcdh19* knockdown clone is smaller in size and more laterally dispersed. The *Pcdh19* knockdown neurons also exhibit a transient increase in the number of neurites at the embryonic stage. Moreover, *Pcdh19* overexpression leads to opposite phenotypes to *Tbr2* deletion—an increase in clonal size and lateral clustering of clonally related neurons. Furthermore, simultaneous expression of PCDH19 largely rescues the defects of clones lacking TBR2, including size, spatial distribution, and preferential synaptic connectivity. We observed more complete rescue of superficial layer neuron production than deep layer neuron production, likely reflecting the timing of PCDH19 expression with regard to TBR2 removal and deep versus superficial layer neuron generation. These results strongly suggest that TBR2 functions through PCDH19 in coordinating the production and precise structural and functional organization of cortical excitatory neurons. It is important to note that, while TBR2 is preferentially expressed at the embryonic stage, PCDH19 is persistently expressed at the postnatal stage and contributes to synapse development. Moreover, the postnatal expression of PCDH19 is reduced in the *Tbr2* mutant cortex, indicating that PCDH19 expression can be indirectly regulated by TBR2, likely via other TBR2 targets. The persistent expression of PCDH19 likely coordinates TBR2 functions in regulating neuronal production at the embryonic stage and neuronal spatial and synaptic organization at the postnatal stage. However, given the temporal difference and distinct nature of the underlying cellular processes, the mechanisms by which PDCH19 governs these processes may be different.

PCDH19 is a non-clustered δ2-type Protocadherin^47^. Previous studies showed that δ-PCDHs can mediate Ca^2+^-dependent hemophilic cell-cell adhesion^47^. Even though their binding strength appears to be much weaker than that of the classic Cadherins, they likely play an important role in intercellular regulation. Consistent with this, PCDH19 has been found to influence neuronal migration in vitro and control cell sorting in the zebrafish optic tectum as well as in the mouse cortex^48–50,56^. Consistent with its role in controlling cell sorting and positioning, we found that removal of PCDH19 or TBR2 causes excessive lateral dispersion of neuronal clones in the cortex. While the precise expression pattern of PCDH19 in individual neuronal clones remains to be determined, our data suggest that PCDH19 functions downstream of TBR2 in controlling fine-scale neuronal positioning in the mammalian cortex. The precise positioning of individual neurons likely involves intra-clonal as well as inter-clonal neuronal interactions that depend on PCDH19 expression.

PCDH19 has also been shown to interact heterotypically with other non-clustered PCDHs and contribute to adhesion specificity in a combinatorial manner^49^. Cells can vary the number of δ-PCDHs expressed, the level of surface expression, and which δ-PCDHs are expressed^57^. This raises the possibility that PCDH19 may directly affect intercellular interaction and synapse development of cortical neurons. Interestingly, PCDH19 expression restores the preferential synaptic connectivity between clonally related excitatory neurons in the absence of TBR2. Notably, human *PCDH19* mutations have been linked to early onset epilepsy and intellectual disability in females^58–60^. Our data show that PCDH19 regulates fine-scale cortical neuronal connectivity, which would contribute to abnormal brain activity and epilepsy in disease conditions.

Structural malformations of the cortex including focal dysplasia, thickening, and abnormal folding have also been observed in human patients with *PCDH19* mutations^61^. In addition to neuronal distribution and synaptic connectivity, our data suggest that PCDH19 also influences neuronal production at the clonal level. Human patients carrying *PCDH19* mutations occasionally exhibit microcephaly^60^. Given that the systematic *Pcdh19* knockout mice did not exhibit any obvious defects in the cortex^56^, the function of PCDH19 on neurogenesis likely relies on its mosaic expression as well. Notably, a recent study showed that PCDH19 in human pluripotent stem cells is localized at the spindle poles during mitosis and co-localizes with N-Cadherin and ZO-1 at the lumen of the neural rosette^62^, indicating that PCDH19 may regulate progenitor cell polarity, organization, and division. PCDH19 likely also influences progenitor-progenitor interaction and consequently cell signaling linked to progenitor behavior regulation. Along this line, PCDH19 removal has recently been shown to promote neural progenitor cell differentiation and neurogenesis (i.e., premature loss of progenitor cell fate)^63^. This is consistent with a reduction in the *Tbr2* mutant clone size that we observed. It is worth noting that TBR2 regulates the expression of other target genes as well^51^, which likely contributes to its function in controlling cortical neurogenesis and neuronal organization.

In summary, our study demonstrates a previously unappreciated function of TBR2 together with PCDH19 in coordinating the production and fine-scale spatial and synaptic organization of excitatory neurons in the cortex. TBR2^+^ IPs play a crucial role in cortical evolution and expansion. While present in the developing dorsal cortex of reptiles, such as turtles, they are relatively scarce and not organized into a discrete SVZ^23^. The abundance and organization of TBR2^+^ IPs has been postulated to drive the radial expansion of the forebrain from a three-layered cortex characteristic of the reptiles to the six-layered neocortex in the mammals^30,64^. The coordinated regulation of increased neurogenesis and precise spatial and synaptic organization of neurons by TBR2 and PCDH19 is likely essential for the structural and functional evolution of the cortex.

## Methods

### Mice

*MADM-11*^*GT*^ and *MADM-11*^*TG*^ mice were produced as previously described^42^. *Emx1-CreER*^*T2*^^41^ and *Tbr2*^*flox/flox*^ (*Tbr2*^*fl/fl*^) mice^39^ were kindly provided by Dr. Nicoletta Tekki-Kessaris and Dr. Anna-Katerina Hadjantonakis, respectively. For MADM labeling, *Emx1-CreER*^*T2*^*;MADM-11*^*GT/GT*^*;Tbr2*^*fl/+*^ mice were crossed with *MADM-11*^*TG/TG*^*;Tbr2*^*fl/fl*^ mice. *Emx1-Cre* mice^43^ were obtained from The Jackson Laboratory (#005628) and crossed with *Tbr2*^*fl/fl*^ to generate cortical *Tbr2* knockout mice. The vaginal plug date was designated as E0, and the birth date was defined as P0. All mouse experiments were performed in accordance with the protocol approved by the Institutional Animal Care and Use Committee of Memorial Sloan Kettering Cancer Center (MSKCC).

### Retrovirus and in utero intraventricular injection

*EGFP, Cre*-T2A-*tdTomato*, *tdTomato*-T2A-*Pcdh19*-T2A-*Cre*, and *Pcdh19*-T2A-*tdTomatto* were inserted into the pUX retroviral vector^65^. Mouse *Tbr2* cDNA was inserted into pCMV vector (Clontech) for luciferase assay. shRNAs against *Pcdh19* were cloned into the pUEG retroviral vector^65^. The *Pcdh19* shRNA sequences were *Pcdh19* shRNA-1, 5′-AATTGACCCACACAGCGGCCT-3′; *Pcdh19* shRNA-2, 5′-AATTATCAACCTCCTGTCGGT-3′; *Pcdh19* shRNA-3, 5′-AACAACACTCCTGGTGCCTAT-3′; *Pcdh19* shRNA-4, 5′-AAGACATCTCTCTCTGCTTCT-3′ (Supplementary Data 3). Retrovirus were produced by co-transfection of retroviral vectors, envelope and packaging plasmids into HEK293T cells followed by ultracentrifugation for viral particles collection^38^. The titer of retrovirus used for clonal labeling was 4~5 × 10^6^ infectious unit per mL, estimated by infecting HEK293T cells with serially diluted retrovirus. Retrovirus with 1% fast green (2.5 mg/mL, Sigma) was injected into the embryonic cerebral ventricle through a beveled, calibrated glass micropipette (Drummond Scientific). For in utero electroporation, 1–1.5 μL of plasmids (2–3 μg/μL) mixed with fast green were injected into the lateral ventricle. Electroporation was carried out using an electroporator (BTX ECM830) (5 pulses; ~40 Volts, 50 ms duration, 950 ms interval). After injection and/or electroporation, the uterus was placed back in the abdominal cavity and the wound was surgically sutured. After surgery, the animal was placed in a recovery incubator under close monitoring until it fully recovered.

### Cell culture and transfection

HEK 293T cells were cultured in Dulbecco’s modified Eagle’s medium (DMEM) supplemented with 10% fetal bovine serum (FBS), non-essential amino acids, and penicillin/streptomycin in a 37 °C, 5% CO_2_ incubator. The cells were plated and cultured overnight before plasmid transfection. Transfection was performed using Lipofectamine 3000 according to the manufacturer’s instructions. Primary neural progenitor cells were isolated from E12 mouse embryonic cortex and cultured in ultra-low attachment surface dishes (Corning) as neurospheres to expand the NPCs^66^. The neurospheres were collected for western blotting and quantitative real-time polymerase chain reaction (RT-qPCR) after 3–4 days in culture. The primary cells dissociated from neurospheres were plated onto dishes coated with Poly-L-ornithine and laminin in neural stem cell basal medium supplemented with 10 ng/ml bFGF and 10 ng/ml EGF and cultured for 24 h before immunocytochemistry.

### Tamoxifen induction

For clone induction, pregnant mice were injected intraperitoneally with tamoxifen dissolved in corn oil at E10, E11, E12, or E13 at a dose of 25–50 mg/kg of body weight. Live embryos were recovered at E19 through cesarean section, fostered, and raised for further analysis. Brains were collected at postnatal stage for further analysis.

### Immunohistochemistry and 3D reconstruction

The mice were perfused with phosphate buffered saline (PBS, pH 7.4), followed by 4% paraformaldehyde (PFA) in PBS. Brains were removed and fixed at 4 °C. Serial coronal sections of individual brains were prepared using a vibratome (Leica) and subjected to immunohistochemistry. Brain sections or cell cultures were blocked in 10% horse serum containing 0.5% Triton X-100 for 1 h at room temperature (RT). Primary antibody incubation was subsequently performed overnight at 4 °C, followed by secondary antibody incubation at RT for 1 h (cultured cells) or 2 h (brain sections). The primary antibodies used were chicken anti-GFP (Aves lab, GFP-1020, 1:1000), rabbit anti-RFP (Rockland, 600–401–379, 1:1000), rabbit anti-PCDH19 (Thermo, PA5-55648, 1:50), rabbit anti-S100 (DAKO, Z0311, 1:500), rabbit anti-OLIG2 (Millipore, ab9610, 1:500), rabbit anti-Histone H3 (CST, 4499, 1:1000), rabbit anti-Cleaved Caspase-3 (CST, 9664, 1:500), rabbit anti-CUX1 (Santa Cruz, sc-13024, 1:100), rat anti-KI67 (Thermo, 14-5698-82, 1:100), rat anti-TBR2 (Thermo, 14-4875-82), rat anti-CTIP2 (Abcam, ab18465, 1:500), goat anti-FOXP2 (Santa Cruz, sc-21069, 1:100), goat anti-BRN2 (Santa Cruz, sc-6029, 1:200), and mouse NEUN (Millipore, MAB377, 1:100).The secondary antibodies were used: goat anti-chicken IgY (H + L) 488 (Thermo, A11039, 1:100), donkey anti-rabbit IgG (H + L) Cy3 (Jackson ImmunoResearch, 711-165-152, 1:1000), goat anti-rat IgG (H + L) 647 (Thermo, A21247, 1:1000), donkey anti-rat IgG (H + L) Cy3 (Jackson ImmunoResearch, 712-165-150, 1:1000), donkey anti-rat IgG (H + L) 488 (Thermo, A21208, 1:1000), donkey anti-goat IgG (H + L) 488 (Thermo, A32814, 1:1000), goat anti-mouse IgG1 488 (Thermo, A21121, 1:1000). Nuclei were counterstained with DAPI (Sigma, D9542, 1:1000). All images were obtained using a confocal microscope (FV1000, Olympus). Z-series images were taken at 1.5–2.5 μm steps and analyzed using FluoView (Olympus), Volocity (ImproVision), Image J (NIH), and Photoshop (Adobe).

For 3D reconstruction, each section was analyzed sequentially from the rostral to the caudal end using Neurolucida and Stereo Investigator (MBF Bioscience). Individual labeled neurons and glia were distinguished based on their morphology and represented as colored dots or stars, respectively. Layer boundaries based on nuclear staining were traced and aligned. Cortical areas were identified using the Allen Brain Atlas. The neuronal position and distribution were analyzed using Neurolucida (MBF Bioscience), MATLAB (MathWorks), and R-Studio (RStudio). The neuronal morphology was further quantified with Sholl analysis using Neurolucida and Stereo Investigator (MBF Bioscience).

### Fluorescence in situ hybridization (FISH)

The FISH was performed on the frozen brain sections using the *Pcdh19* probe (Mm-*Pcdh19*-C1, 417361, Advanced Cell Diagnostics) and RNAscope Fluorescent Multiplex Reagent Kit (Advanced Cell Diagnostics, 320850) according to the vendor protocols (Advanced Cell Diagnostics 320535 and 320293-UM). In brief, the mice were perfused with RNase free PBS and 4% PFA in RNase free PBS. Brains were removed and fixed overnight with 4% PFA in RNase free PBS at 4 °C. After dehydration with 30% sucrose in RNase free PBS for 48 h at 4 °C, the brains were embedded in OCT compound (Sakura, 4583) and frozen. The frozen brains were cut coronally at 10 μm thickness. The brain sections were boiled with target retrieval reagent (Advanced Cell Diagnostics, 322000) for 5–6 min at 100 °C, after wash with distilled water (Thermo, 10977023) and 100% ethanol (Fisher, BP28184) then incubated with protease IV (Advanced Cell Diagnostics, 322340) for 30 min at 40 °C. After wash with distilled water (Thermo, 10977023), *Pcdh19* probe was added to sections then incubated for 2 h at 40 °C. Sequentially incubated with AMP1 (30 min, 40 °C), AMP2 (15 min, 40 °C), AMP3 (30 min, 40 °C), AMP4-B (15 min, 40 °C), sections were washed with the wash buffer (Advanced Cell Diagnostics, 310091) between two incubation. Nuclei were counterstained with DAPI. All images were obtained using a confocal microscope (FV1000, Olympus). Z-series images were taken at 1.5 μm steps and analyzed using FluoView (Olympus), Volocity (ImproVision), Image J (NIH), and Photoshop (Adobe).

### Real-time polymerase chain reaction (RT-PCR) analysis

Total RNA was extracted from brain tissue or cultured cells using the RNeasy Micro Kit (QIAGEN), according to the manufacturer’s directions. Complementary DNA was reverse-transcribed from total RNA samples using the QuantiTect Rev. Transcription Kit (QIAGEN). Quantitative real-time PCR was performed using the PowerUp™ SYBR® Green Master Mix (Thermo) in 20 μL of reaction mixture on a StepOnePlus™ Real-Time PCR system (Applied Biosystems). The primer sequences for *β-Actin* (Forward, 5′-GGCTGTATTCCCCTCCATCG-3′; Reverse, 5′-CCAGTTGGTAACAATGCCATGT-3′), *Tbr2* (Forward, 5′-CCACGTCTACCTGTGCAACC-3′; Reverse, 5′-GAAATCTCCTGCCTCATCCA-3′), and *Pcdh19* (Forward, 5′-TGGCAATCAAATGCAAGCGT-3′; Reverse, 5′-ACCGAGATGCAATGCAGACA-3′) were previously published (Supplementary Data 3)^56,67^. The relative amount of each mRNA was determined by the 2^−ΔΔCT^ method^68^. All qRT-PCR studies were repeated at least three times in triplicate for each sample.

### Chromatin immunoprecipitation qPCR (ChIP-qPCR)

Chromatin immunoprecipitation (ChIP) was performed using ChIP kit according to the manufacturer’s instructions (Abcam). Briefly, E14 mouse embryonic neocortex was dissociated by incubation with Accutase (Thermo). The primary cells were fixed in 1% formaldehyde and cross-linked cell lysates were sheared by sonication to generate chromatin fragments with an average length of 400–850 bp. Complexes containing the target were then immunoprecipitated using the specific antibody (rabbit anti-TBR2, Abcam, ab23345; rabbit anti-IgG, Abcam, ab171870) overnight at 4 °C. On the next day, the antibody-chromatin complexes were incubated with protein A beads for 1 h at 4 °C. After extensive steps, the genomic DNA extraction was purified. Samples were subjected to qPCR using specific primers on a StepOnePlus™ Real-Time PCR system (Applied Biosystems). The specific primers for real-time qPCR detection used were: region1-F: 5′-GCAAACACACCCTGATTTCCC-3′, region1-R: 5′-TGAGCTGGGATGGATCAACA-3′; region2-F: 5′-TCCAGGAAGCAATCTGGTGA-3′, region2-R: 5′-AGCTGGGGCTGTTAGTTTCA-3′ (Supplementary Data 3). Fold enrichment was calculated as described previously^69^.

### Luciferase assays

The TBR2 binding fragments (region 1 and region2) upstream and downstream of *Pcdh19* were PCR amplified and cloned into the pGL3 luciferase reporter vector (Promega). The amplification primer sequences for the two regions were Region1-F: 5′-TTGTGCTGGCTATTTGCCTT-3′, Region1-R: 5′-TTATTACCTTTCAGCTTGTT-3′; Region2-F: 5′-ACTCTACATTAGCAGGTTTG-3′, Region2-R: 5′-TATGACAATTGAACATTAAT-3′ (Supplementary Data 3). HEK293T cells were seeded into 96-well plates and cotransfected with the luciferase reporter vectors and *Tbr2* expression plasmid. Around 48 h after transfection, cells were harvested for luciferase assay using the Dual-Glo Luciferase Reporter Assay System (Promega) according to the manufacturer’s instructions.

### Microarray analysis

FACS isolation, RNA extraction, and microarrays were performed as described previously^70^. Data for *Tbr2* cKO mice include five biological replicates for E13 and six for E16 (from three different litters) (GEO accession number: GSE121180). The data from corresponding control littermates were published previously^70^ (GEO accession number GSE45450). Background-subtracted signal intensity for the detected probes (detection *P* < 0.05) was used for analysis. Probes detected in all arrays were considered during analysis. Further, outlier probes were removed and data were quantile-normalized. Differential expression was determined using two-way ANOVA. FDR (Benjamini-Hochberg) method of multiple testing correction was used for determining adjusted *p*-values. Probes for which the differences between groups were >1.5-fold and with adjusted *p*-value < 0.05 were considered differentially expressed.

DAVID (the Database for Annotation, Visualization and Integrated Discovery)^71^ version 6.7 was used for overrepresentation analysis. The common set of differentially expressed probes of E13 (Mut versus Ctrl) and E16 (Mut versus Ctrl) were used as input. The entire probe-set used for statistical analysis were listed as background. Benjamini adjusted *p*-value < 0.05 was considered significant.

### Western blotting assay

Protein was isolated from cultured cells with RIPA lysis buffer supplemented with protease inhibitor (Roche). The homogenates were centrifuged at 17,000 × *g* for 20 min at 4 °C. The supernatants were collected, and protein concentration was measured with the BCA kit (Thermo). Protein samples were run on 10% SDS-PAGE gels (BioRad) and transferred to PVDF membranes (Millipore). The membranes were blocked with 5% bovine serum albumin (BSA) dissolved in Tris-buffered saline (TBS) containing 0.05% Tween 20 for 1 h at RT. Membranes were incubated with primary antibody at 4 °C overnight, followed by incubation with secondary antibodies for 1 h at RT. The primary antibodies used in western blotting were rabbit anti-PCDH19 (Millipore, ABT318, 1:2000), rabbit anti-GAPDH (Santa Cruz, sc-32233, 1:1000), rabbit anti-Histone H3 (CST, 4499, 1:5000), and mouse anti-β-ACTIN (Proteintech Group, 60008–1, 1:5000). Secondary antibodies were rabbit or mouse HRP-conjugated secondary antibody.

### Electrophysiology

Embryos that received retroviral injections were delivered naturally. Brains were removed at various times after birth, and acute cortical slices (350 μm) were prepared in artificial cerebrospinal fluid (ACSF) containing 126 mM NaCl, 3 mM KCl, 1.25 mM KH_2_PO_4_, 1.3 mM MgSO_4_, 3.2 mM CaCl_2_, 26 mM NaHCO_3_ and 10 mM glucose, bubbled with 95% O_2_ and 5% CO_2_, with a vibratome (Leica) at 4 °C. Slices were allowed to recover in an interface chamber at 32 °C for at least 1 h and were then kept at RT before being transferred to a recording chamber containing ACSF at 34 °C. An IR-DIC microscope (Olympus) equipped with epi-fluorescence illumination, a charge-coupled device camera and two water immersion lenses (10× and 60×) were used to visualize and target the recording electrodes to EGFP- or tdTomato-expressing clonally related excitatory neurons and their nearby non-fluorescently labeled excitatory neurons. Glass recording electrodes (~10 MΩ resistance) were filled with an intracellular solution consisting of 130 mM potassium gluconate, 6 mM KCl, 2 mM MgCl2, 0.2 mM EGTA, 10 mM HEPES, 2.5 mM Na2ATP, 0.5 mM Na2GTP, 10 mM potassium phosphocreatine, 0.3% neurobiotin (Vector Lab), and/or Alexa Fluor 488 hydrazide (Thermo, if necessary) (pH 7.25 and 295 mOsmol per kg solution). Recordings were collected and analyzed using an Axon Multiclamp 700B amplifier and pCLAMP 10 software (Molecular Devices).

In quadruple whole-cell recordings (P14–25 animals), synapses were assessed by three brief (5 ms) high suprathreshold (600–1000 pA) depolarization current injections separated by 500 msec (i.e., 2 Hz) and one long (200 ms) high suprathreshold (600–1000 pA) depolarization current injected into one of the four neurons sequentially and the responses of all neurons were monitored. For chemical synapse detection, the neuron that received current injection was maintained under current clamp mode and the other neurons were maintained under voltage clamp mode at −70 mV. The criterion was that the average postsynaptic current was larger than 0.5 pA within 1–5 ms after the peak of the presynaptic action potential.

In whole-cell patch-clamp recording experiments, slices were fixed in 4% PFA in phosphate buffered saline (PBS, pH 7.4) after the recordings were completed, and the morphology of recorded neurons, that were loaded with neurobiotin, was later visualized with Alexa Fluor 647- or Alexa Fluor 488-conjugated streptavidin (Thermo) using a confocal laser scanning microscope (Olympus FV1000). Z-series images were taken at 1.5–2.5 μm steps and analyzed using FluoView (Olympus) and Photoshop (Adobe).

### Statistical analysis

Significance was determined using the two-tailed unpaired Student’s *t*-test, chi-square test, one-way ANOVA followed by a Tukey’s test for post hoc multiple comparisons, and two-way ANOVA by GraphPad Prism 7 software. Differences were considered statistically significant at *P < 0.05, **P < 0.01, ***P < 0.001, and ****P < 0.0001. Data are presented as mean ± SEM.

### Reporting summary

Further information on research design is available in the Nature Research Reporting Summary linked to this article.

## Supplementary information

Supplementary information

Description of Additional Supplementary Files

Supplementary Data 1

Supplementary Data 2

Supplementary Data 3

Reporting Summary

## Data Availability

The data that support the findings of this study are available from the corresponding author upon reasonable request.
