# Supplementary material for: TBR2 coordinates neurogenesis expansion and precise microcircuit organization via Protocadherin 19 in the mammalian cortex
Source: Nat Commun. 2019 Sep 2;10:3946. doi: 10.1038/s41467-019-11854-x (PMC6718393; doi:10.1038/s41467-019-11854-x)
Supplement: Supplementary file 1 — Supplementary information [file 41467_2019_11854_MOESM1_ESM.pdf]

Supplementary information

TBR2 coordinates neurogenesis expansion and precise microcircuit organization via Protocadherin 19 in the mammalian cortex

Lv et al

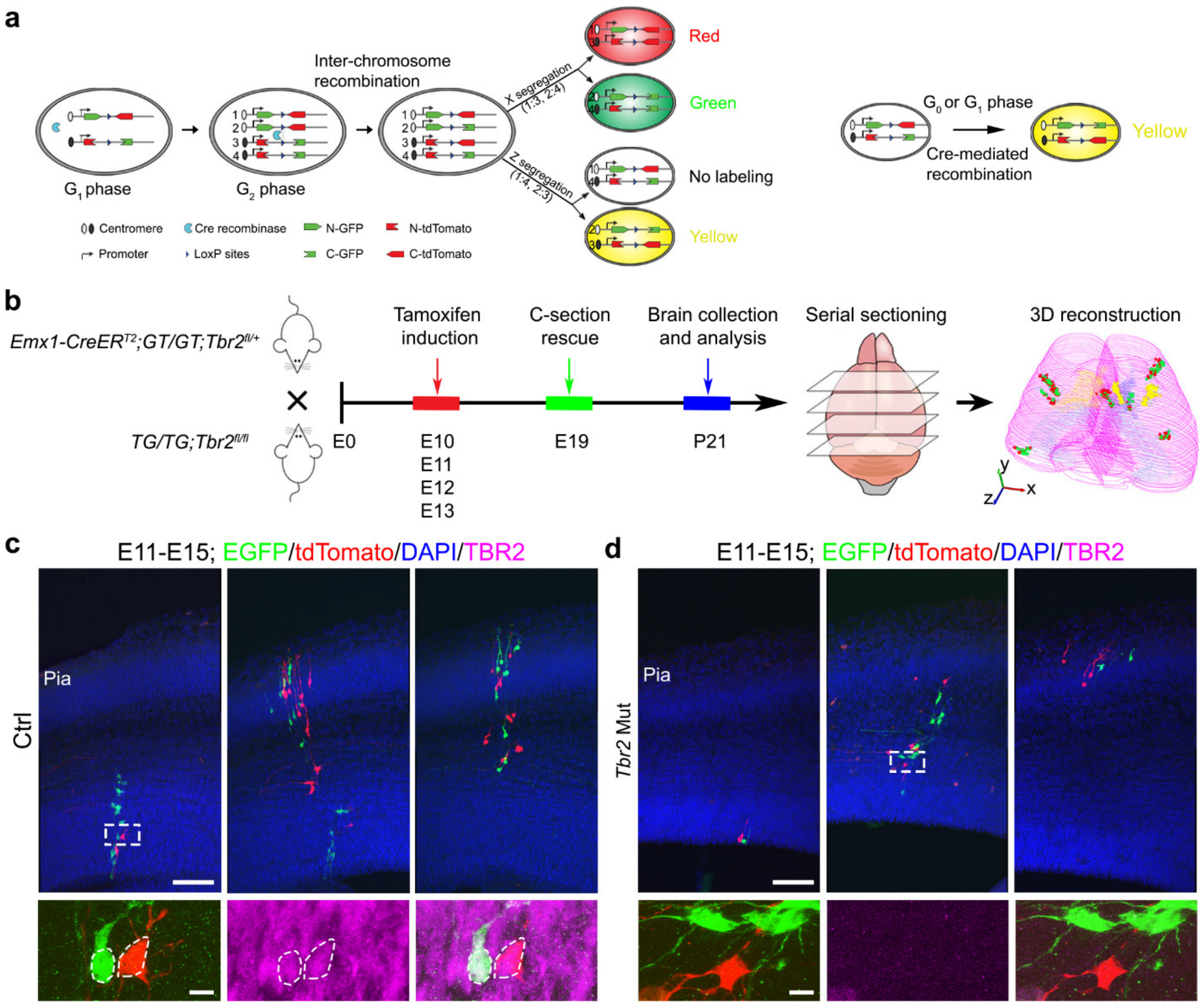

Supplementary Fig. 1: Outline of MADM-based clonal analysis of neocortical excitatory neuron production and organization in the absence of TBR2. a, Schematic diagram of MADM labeling strategy. **b,** Experimental procedure of the MADM-based clonal analysis in the control and *Tbr2* mutant cortex. **c,d,** Representative confocal images of the E15 control (c) and *Tbr2* Mut (d) cortices treated with tamoxifen at E11 and stained for TBR2 (purple) and counter-stained with DAPI (blue). High-magnification images of MADM-labeled cells in the SVZ (broken lines) are shown at the bottom. Note the effective removal of TBR2 in the *Tbr2* Mut cortices. Scale bars: 50 µm (top) and 5 µm (bottom).

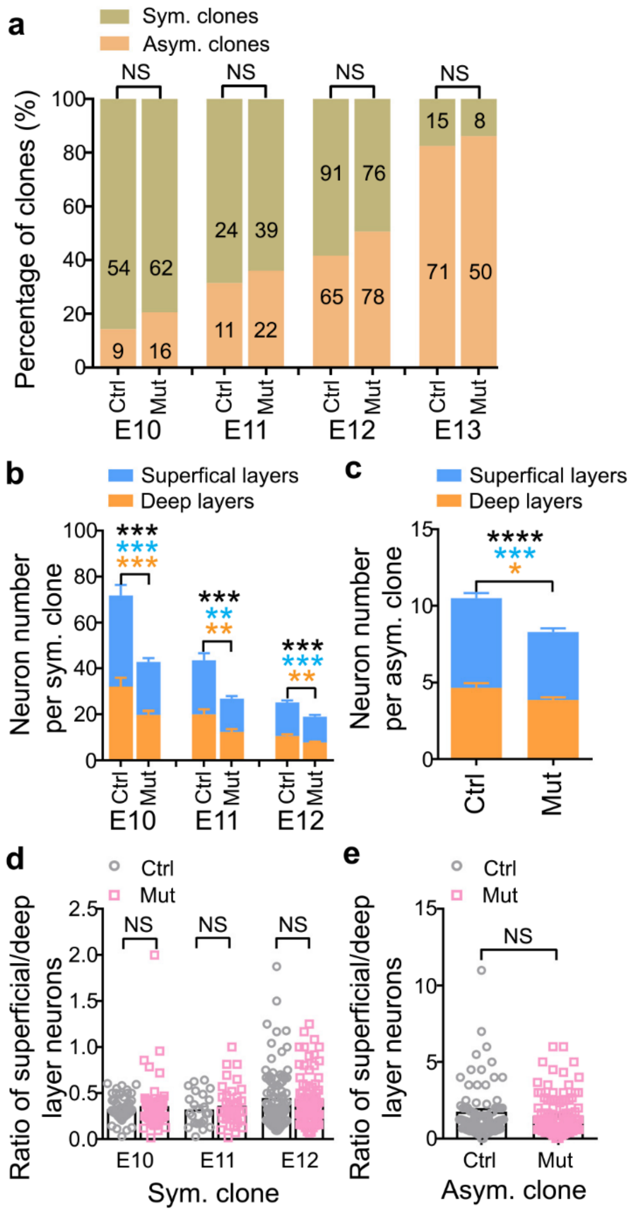

Supplementary Fig. 2: TBR2 removal leads to a reduction in the neuronal number in both symmetric and asymmetric clones. a, Percentage of symmetric versus asymmetric clones labeled at different embryonic stages in the Ctrl and *Tbr2* Mut cortices. The numbers of clones are shown in the graph. Data are presented as mean ± SEM (NS, not significant; chi-square test). **b,c,** Reduction in the neuronal number in *Tbr2* Mut symmetric (b) (E10: Ctrl, $n = 54$; Mut, $n = 62$; E11: Ctrl, $n = 24$; Mut, $n = 39$; E12: Ctrl, $n = 91$; Mut, $n = 76$) and asymmetric (c) (Ctrl, $n = 85$; Mut, $n = 116$) clones labeled at different embryonic stages compared with Ctrl. **d,e,** The ratio of superficial (2-4) versus deep (5-6) layer neuronal numbers in the Ctrl and *Tbr2* Mut symmetric (d) and asymmetric (e) clones. Data are presented as mean ± SEM (*$P < 0.05$; **$P < 0.01$; ***$P < 0.001$; ****$P < 0.0001$; NS, not significant; unpaired Student's t-test).

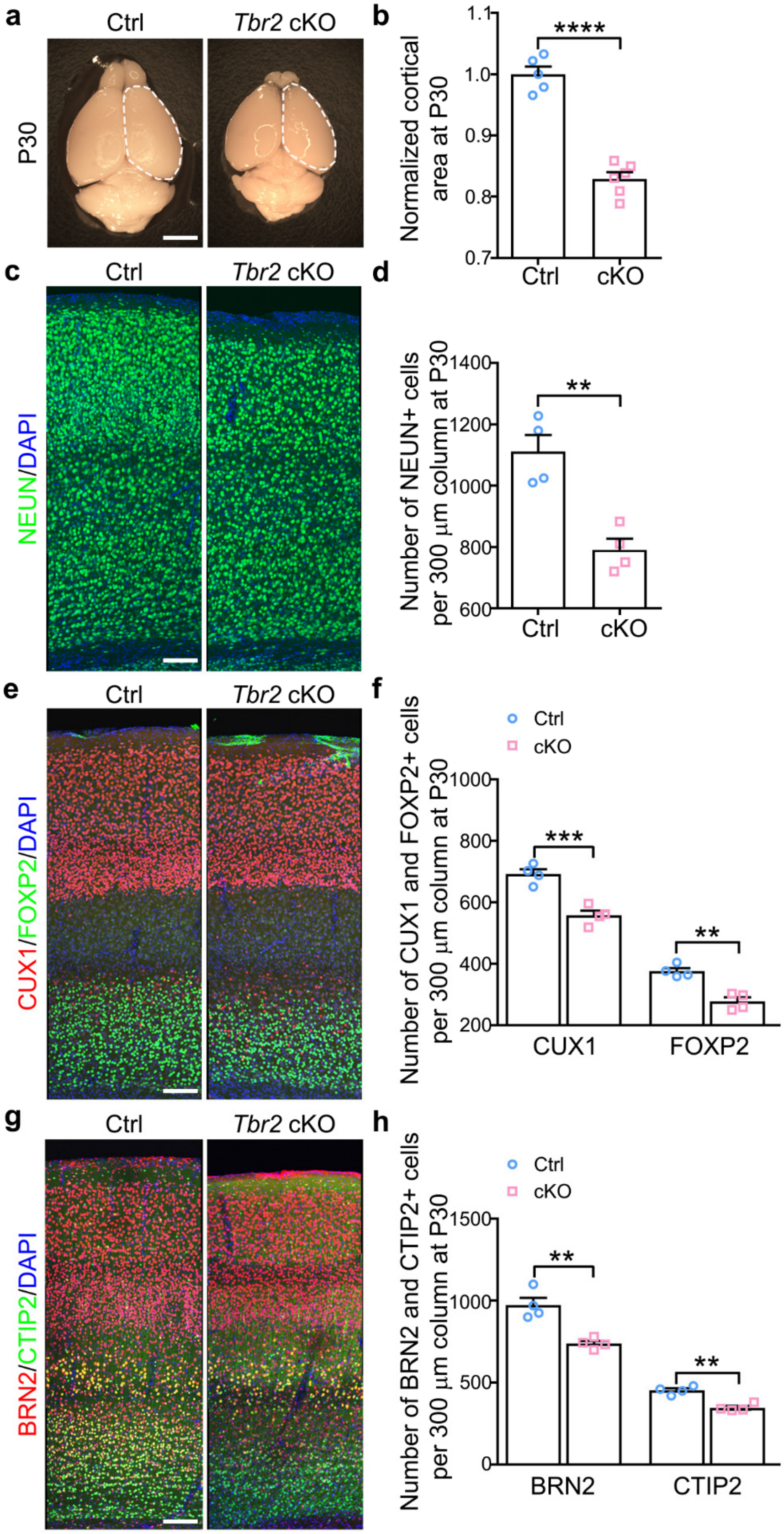

a
Ctrl
Tbr2 cKO
P30
b
****
Normalized cortical area at P30
1.1
1.0
0.9
0.8
0.7
Ctrl
cKO
c
NEUN/DAPI
d
**
Number of NEUN+ cells per 300 μm column at P30
1400
1200
1000
800
600
e
CUX1/FOXP2/DAPI
f
Ctrl
cKO
***
**
Number of CUX1 and FOXP2+ cells per 300 μm column at P30
1000
800
600
400
200
CUX1
FOXP2
g
BRN2/CTIP2/DAPI
h
**
**
Number of BRN2 and CTIP2+ cells per 300 μm column at P30
1500
1000
500
0
BRN2
CTIP2

Supplementary Fig. 3: TBR2 removal leads to a loss of both deep and superficial layer neurons in the cortex. a, Representative whole mount images of the wild type control (Ctrl) and *Tbr2* conditional knockout (cKO) brains at P30. **b,** Quantification of the cortical area of P30 Ctrl and *Tbr2* cKO brains (Ctrl, n = 5; cKO, n = 6). **c,** Representative confocal images of the Ctrl and *Tbr2* cKO cortices at P30 stained for NEUN (green) and DAPI (blue). **d,** Quantification of the number of $NEUN^+$ cells per 300 μm radial column at P30 (Ctrl, n = 4; cKO, n = 4). **e,** Representative confocal images of the Ctrl and *Tbr2* cKO cortices at P30 stained for CUX1 (green), FOXP2 (red), and DAPI (blue). **f,** Representative confocal images of the Ctrl and *Tbr2* cKO cortices at P30 stained for BRN2 (green), CTIP2 (red), and DAPI (blue). **g,** Quantification of the number of $CUX1^+$ and $FOXP2^+$ cells per 300 μm radial column at P30 (Ctrl, n = 4; cKO, n = 4). **h,** Quantification of the number of $BRN2^+$ and $CTIP2^+$ cells per 300 μm radial column at P30 (Ctrl, n = 4; cKO, n = 4). Scale bars: 2 mm (a) and 100 μm (c, e, and g). Data are presented as mean ± SEM (**P < 0.01; ***P < 0.001; ****P < 0.0001; unpaired Student's t-test).

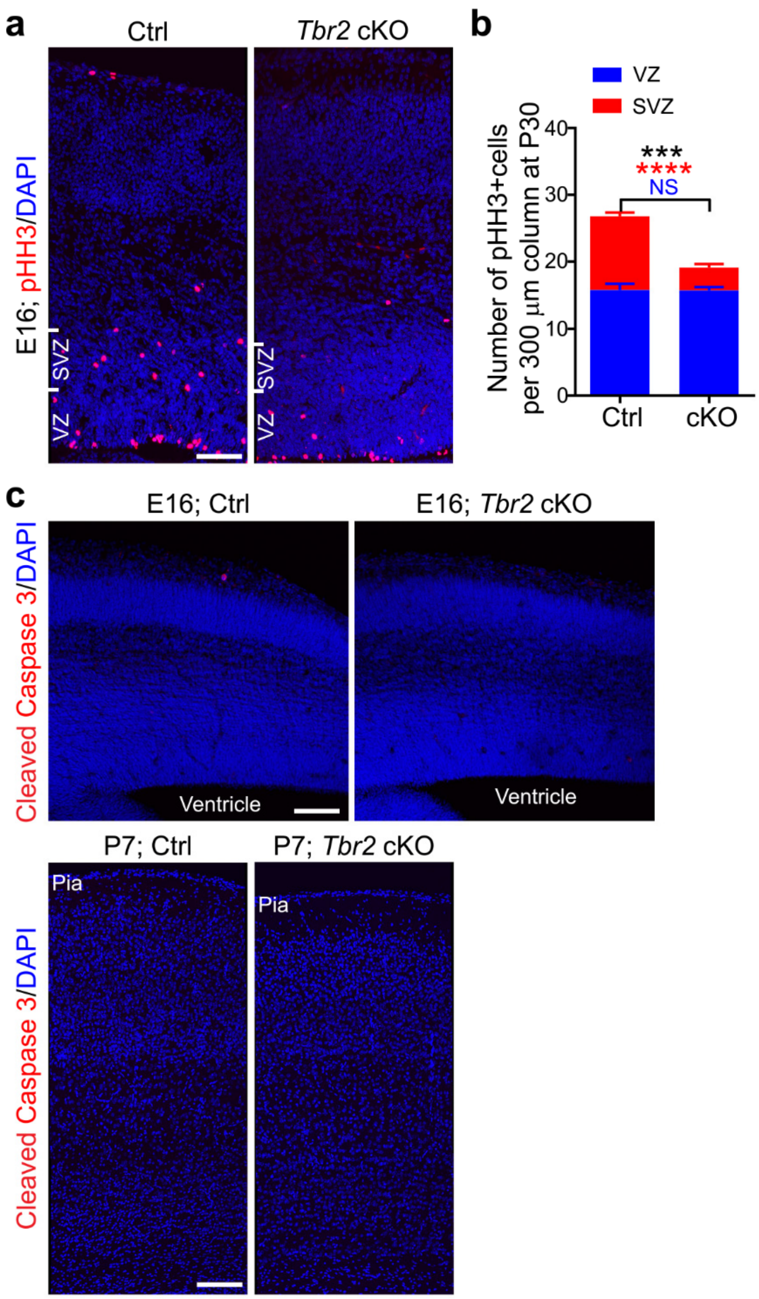

Supplementary Fig. 4: TBR2 removal leads to a loss of mitotic cells in the SVZ with no obvious change in cell death. a, Representative confocal images of the Ctrl and *Tbr2* cKO cortices at E16 stained for phosphorylated Histone H3 (pHH3, red) and DAPI (blue). **b,** Quantification of the number of $pHH3^+$ cells per 300 µm radial column at E16 (Ctrl, *n* = 5; cKO, *n* = 7). **c,** Representative confocal images of the Ctrl and *Tbr2* cKO cortices at E16 (top) and P7 (bottom) stained for Cleaved Caspase 3 (red) and DAPI (blue). Scale bars: 50 µm (a) and 100 µm (c). Data are presented as mean ± SEM (****P* < 0.001; *****P* < 0.0001; NS, not significant; unpaired Student's t-test).

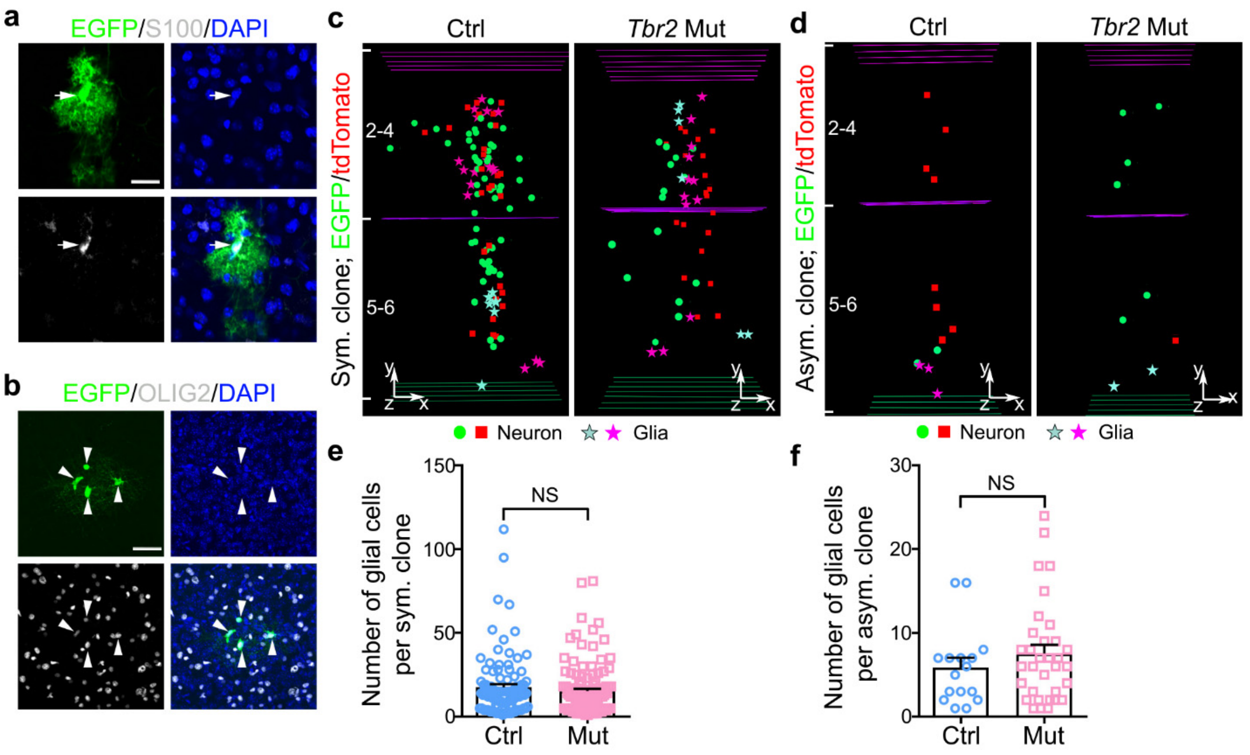

Supplementary Fig. 5: TBR2 removal does not affect gliogenesis. a,b, Representative confocal images of the MADM-labeled cells (arrows, a; arrowheads, b) stained for S100 (a, white), a well-characterized astrocyte marker, or OLIG2 (b, white), a well-characterized oligodendrocyte marker. Scale bars: 20 µm (a) and 40 µm (b). **c,d,** 3D reconstruction images of representative Ctrl and *Tbr2* Mut symmetric (c) and asymmetric (d) clones with glial cells. Different colored lines indicate the layer boundaries. **e,f,** Quantification of the glial cell number in Ctrl and *Tbr2* Mut symmetric (e; Ctrl, n = 100; Mut, n = 125) and asymmetric (f; Ctrl, n = 16; Mut, n = 33) clones. Data are presented as mean ± SEM (NS, not significant; unpaired Student's t-test).

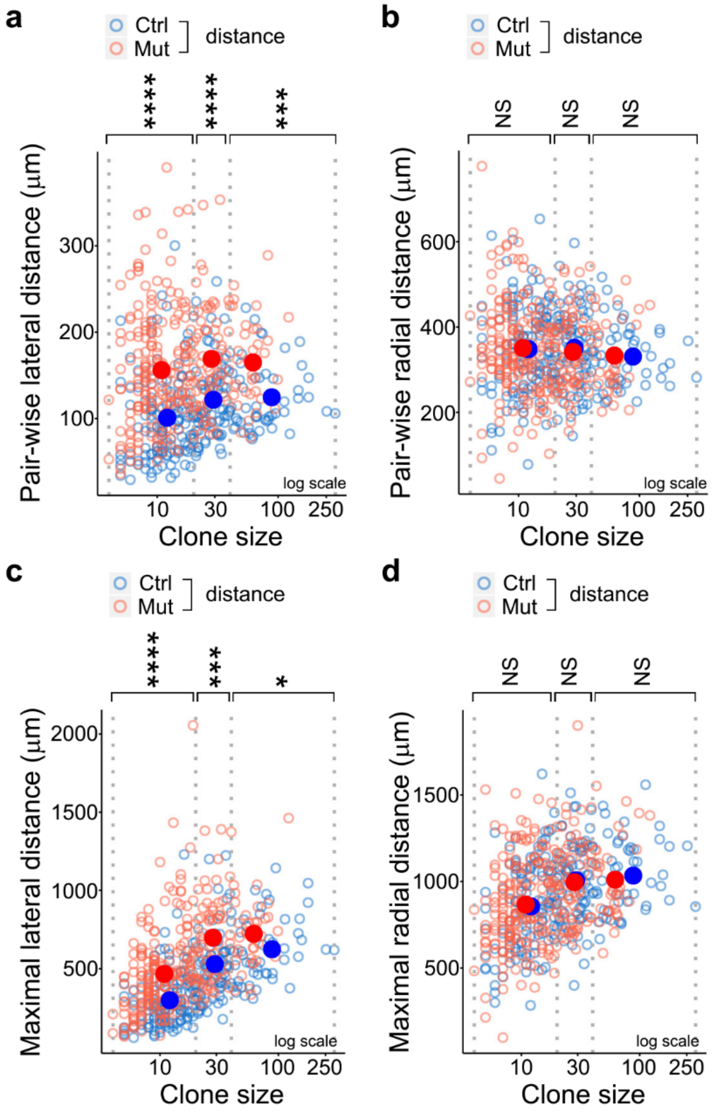

Supplementary Fig. 6: Similar increase in the pair-wise and maximal lateral distances in *Tbr2* Mut clones with different numbers of neurons. a-d, Quantification of the pair-wise (a,b) and maximal (c,d) lateral and radial distances of Ctrl and *Tbr2* Mut (symmetric and asymmetric) clones with regard to the number of neurons in individual clones (Ctrl, n = 254; Mut, n = 293). The clones were divided into three groups based on their clone sizes (broken lines: group 1, 1-20; group 2, 21-40; group 3, >40). Each open circle represents an individual clone and the filled circles indicate the means of distance and clone size of each group. Note the consistent increase in the pair-wise and maximal lateral distances in the *Tbr2* Mut clone compared with the Ctrl clone across the three groups (* $P < 0.05$; *** $P < 0.001$; **** $P < 0.0001$; NS, not significant; unpaired Student's t-test). Data are presented as mean ± SEM.

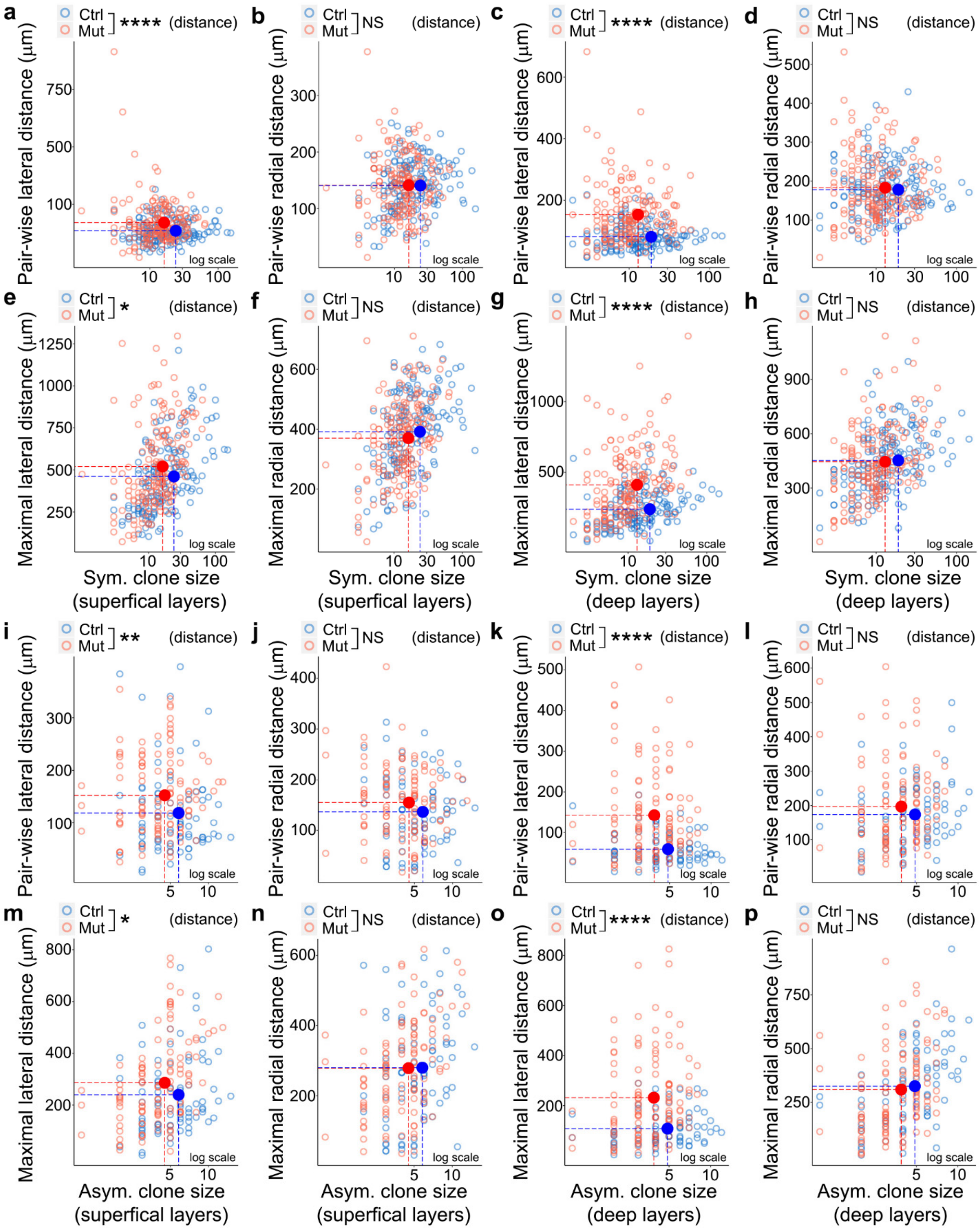

Supplementary Fig. 7: TBR2 regulates spatial organization of clonally related neurons in both superficial and deep layers. a-h, Quantification of the pair-wise (a-d) and maximal (e-h) lateral and radial distances between neurons in Ctrl and *Tbr2* Mut symmetric clones of

superficial (a,b,e,f) and deep layers (c,d,g,h) (Ctrl, n = 169; Mut, n = 177). **i-p,** Quantification of the pair-wise (i-l) and maximal (m-p) lateral and radial distances between neurons in Ctrl and *Tbr2* asymmetric clones of superficial (i,j,m,n) and deep layers (k,l,o,p) (Ctrl, n = 85; Mut, n = 116). Data are presented as mean ± SEM (*$P < 0.05$, **$P < 0.01$, ****$P < 0.0001$, and NS, not significant; unpaired Student's t-test).

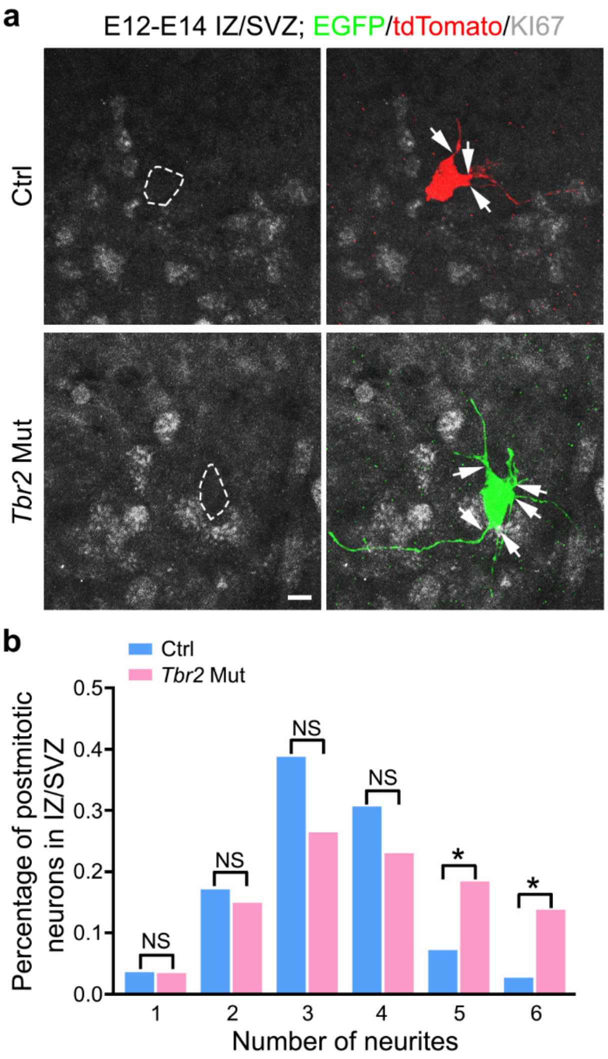

Supplementary Fig. 8: Increase in the number of neurites in post-mitotic neurons of the *Tbr2* Mut clones. a, Confocal images of representative E14 Ctrl and *Tbr2* Mut MADM clones labeled by TM administration at E12 and stained for KI67 (white). Note the post-mitotic neurons expressing EGFP (green) or tdTomato (red) but not KI67. Scale bars: 10 µm. **b,** Quantification of the percentage of post-mitotic neurons possessing different numbers of neurites in the Ctrl and *Tbr2* Mut clones (Ctrl, n = 111 neurons from 38 clones; Mut, n = 87 neurons from 30 clones). Data are presented as mean ± SEM (*$P < 0.05$ and NS, not significant; chi-square test).

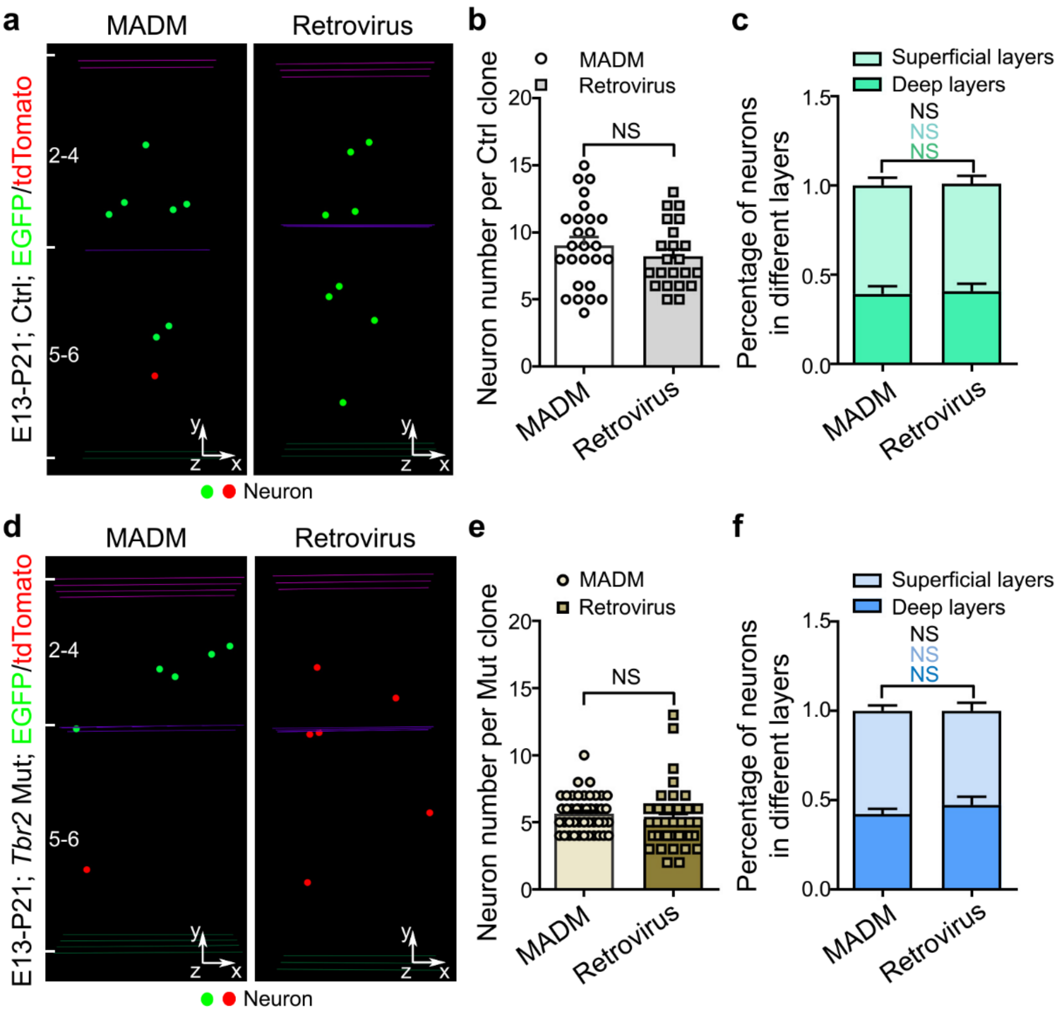

Supplementary Fig. 9: Clones labeled by low-titer retrovirus infection and MADM exhibit similar size and laminar distribution. a, 3D reconstruction images of representative P21 control clones labeled by MADM or low-titer retrovirus infection at E12-13. **b,** Quantification of the number of neurons in the control clone labeled by MADM and low-titer retrovirus infection (MADM, n = 26; Retrovirus, n = 22). **c,** Quantification of the percentage of neurons in superficial and deep layers in the control clone labeled by MADM and low-titer retrovirus infection (MADM, n = 26; Retrovirus, n = 22). **d,** 3D reconstruction images of representative P21 *Tbr2* Mut clones labeled by MADM or low-titer retrovirus infection at E12-13. **e,** Quantification of the number of neurons in the *Tbr2* Mut clone labeled by MADM and low-titer retrovirus infection (MADM, n = 46; Retrovirus, n = 37). **f,** Quantification of the percentage of neurons in superficial and deep layers in the *Tbr2* Mut clone labeled by MADM and low-titer retrovirus infection (MADM, n = 46; Retrovirus, n = 37). Data are presented as mean ± SEM (NS, not significant; unpaired Student's t-test).

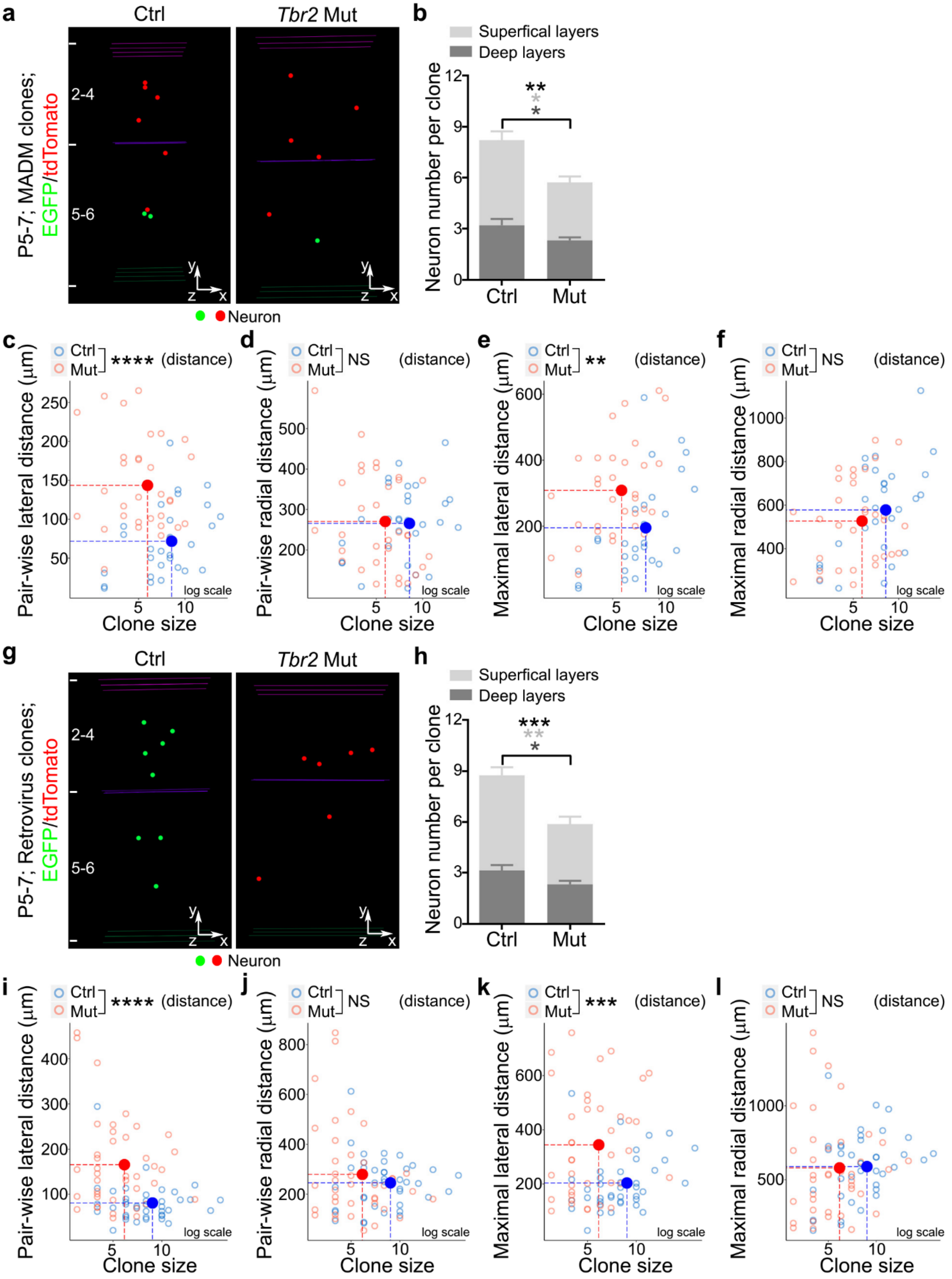

a
Ctrl
Tbr2 Mut
P5-7; MADM clones; EGFP/tdTomato
2-4
5-6
y
z
x
Neuron
b
Superfical layers
Deep layers
Neuron number per clone
**
*
*
Ctrl
Mut
c
Ctrl
Mut
**** (distance)
Pair-wise lateral distance (µm)
Clone size
log scale
d
NS
(distance)
Pair-wise radial distance (µm)
e
** (distance)
Maximal lateral distance (µm)
f
NS
(distance)
Maximal radial distance (µm)
g
P5-7; Retrovirus clones; EGFP/tdTomato
h
***
**
*
i
**** (distance)
j
NS
k
*** (distance)
l
NS

Supplementary Fig. 10: TBR2 removal causes a reduction in neuronal output of individual RGPs and lateral dispersion of clonally related excitatory neurons at P5-7. a, 3D reconstruction images of P5-7 Ctrl and *Tbr2* Mut of excitatory neuron clones labeled by tamoxifen treatment at E12-E13. **b,** Quantification of the number of neurons in Ctrl (n = 29) or *Tbr2* Mut (n = 32) clones. **c,d,** Quantification of the pair-wise lateral and radial distances between neurons in Ctrl (n = 29) or *Tbr2* Mut (n = 32) clones. **e,f,** Quantification of the maximal lateral and radial distances between neurons in Ctrl (n = 29) or *Tbr2* Mut (n = 32) clones. **g,** 3D reconstruction images of P5-7 Ctrl and *Tbr2* Mut excitatory neuron clones labeled by low-titer retrovirus infection at E12-13. **h,** Quantification of the number of neurons in Ctrl (n = 39) or *Tbr2* Mut (n = 40) clones. **i,j,** Quantification of the pair-wise lateral and radial distances between neurons in Ctrl (n = 39) or *Tbr2* Mut (n = 40) clones. **k,l,** Quantification of the maximal lateral and radial distances between neurons in Ctrl (n = 39) or *Tbr2* Mut (n = 40) clones. Data are presented as mean ± SEM (*P < 0.05, **P < 0.01, ***P < 0.001, ****P < 0.0001, and NS, not significant; unpaired Student's t-test).

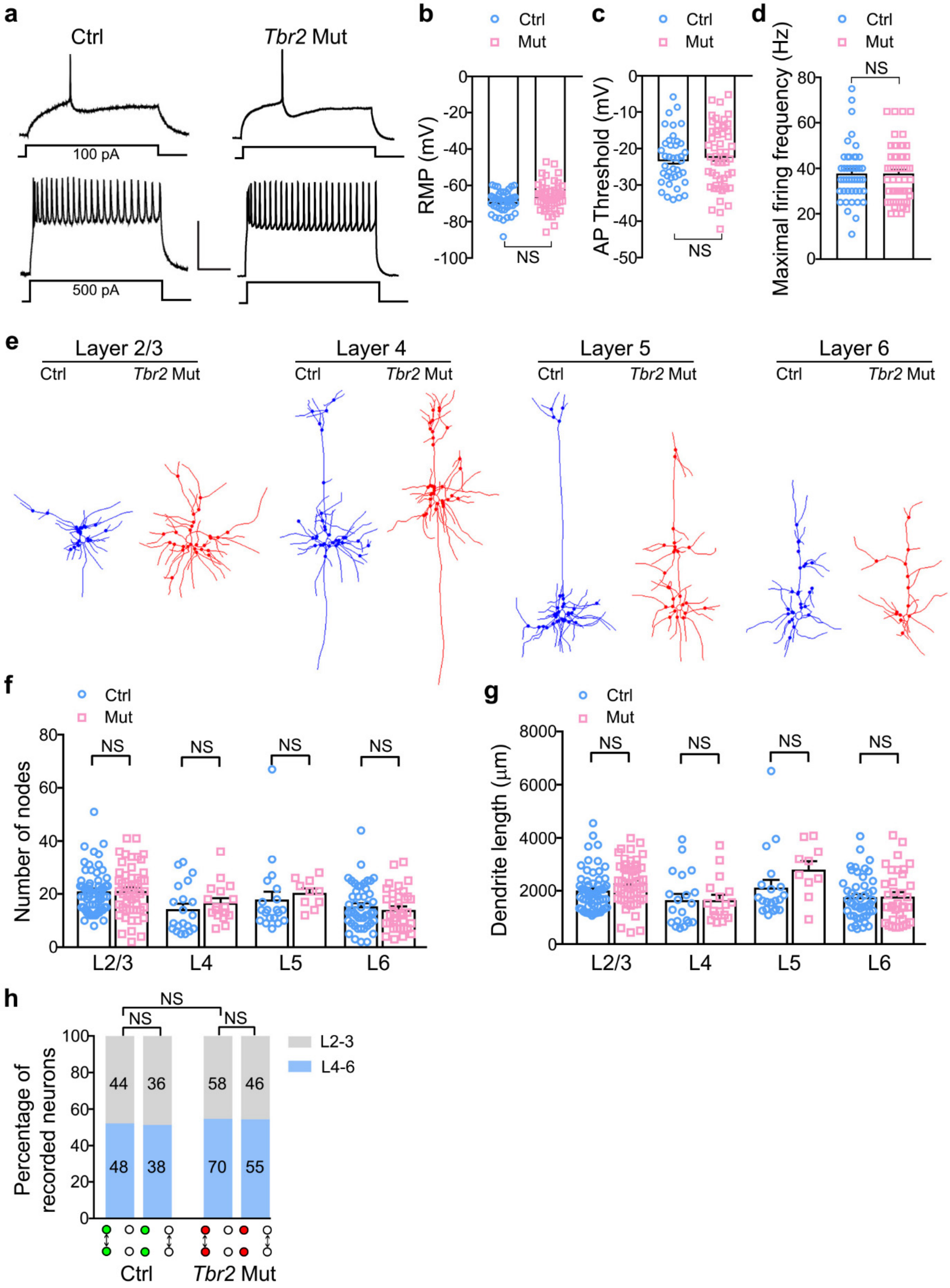
a
Ctrl
Tbr2 Mut
100 pA
500 pA
b
Ctrl
Mut
RMP (mV)
NS
c
AP Threshold (mV)
NS
d
Maximal firing frequency (Hz)
NS
e
Layer 2/3
Layer 4
Layer 5
Layer 6
Ctrl
Tbr2 Mut
f
Number of nodes
NS
L2/3
L4
L5
L6
g
Dendrite length (μm)
h
Percentage of recorded neurons
L2-3
L4-6
44
36
58
46
48
38
70
55
Ctrl
Tbr2 Mut

Supplementary Fig. 11: TBR2 removal does not impair cortical neuronal membrane properties and morphology. a, Representative sample traces of the responses of excitatory neurons to somatic current injections in Ctrl and *Tbr2* Mut neurons. Scale bars: 50 mV and 200 msec. **b-d,** Summary of the resting membrane potential (RMP) (b, Ctrl, n = 45; Mut, n = 55), threshold for firing action potential (c, Ctrl, n = 40; Mut, n = 53), and maximum firing frequency (d, Ctrl, n = 46; Mut, n = 55) of Ctrl and *Tbr2* Mut excitatory neurons. The numbers of recorded neurons are shown in the graph. **e,** Representative reconstructed morphologies of Ctrl and *Tbr2* Mut neurons in different layers. **f,g,** Quantification of the neurite branch number (f) and length (g) of Ctrl and *Tbr2* Mut neurons in different layers (L2/3, Ctrl, n = 62; Mut, n = 50; L4, Ctrl, n = 20; Mut, n = 17; L5, Ctrl, n = 20; Mut, n = 10; L6, Ctrl, n = 47; Mut, n = 35). Data are presented as mean ± SEM (NS, not significant; unpaired Student's t-test). **h,** Quantification of the number of the recorded Ctrl and *Tbr2* Mut neurons in different cortical layers. Data are presented as mean ± SEM (NS, not significant; chi-square test).

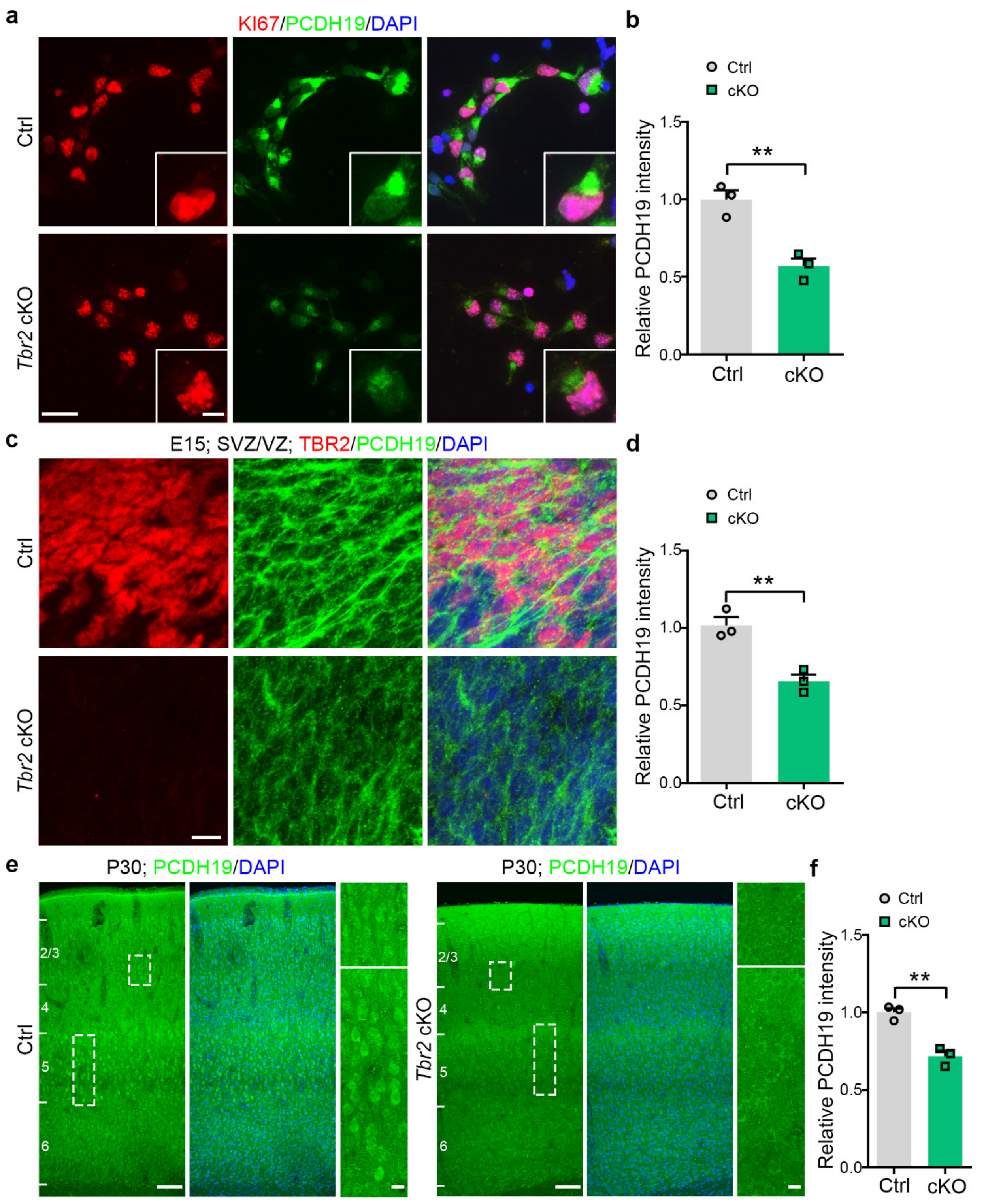

Supplementary Fig. 12: TBR2 removal leads to a reduction in PCDH19 expression at the embryonic and postnatal cortices. a, Representative images of cultured Ctrl and *Tbr2* cKO cortical neural progenitor cells stained for KI67 (red), PCDH19 (green), and with DAPI (blue). High magnification images are shown in insets. Note the decrease in PCDH19 expression in the

Tbr2 cKO cortical neural progenitors compared with Ctrl. Scale bars: 50 μm and 5 μm. **b,** Quantification of the relative PCDH19 staining intensity in Ctrl and *Tbr2* cKO cortical neural progenitors (n = 3 for each genotype). **c,** Representative images of E15 control and *Tbr2* cKO SVZ/VZ stained for TBR2 (red), PCDH19 (green), and counter-stained with DAPI (blue). Note the decrease in PCDH19 expression in the *Tbr2* cKO SVZ/VZ compared with the control. Scale bar: 10 μm. **d,** Quantification of the relative PCDH19 staining intensity in the control and *Tbr2* cKO SVZ/VZ (n = 3 for each genotype). **e,** Representative images of P30 control and *Tbr2* cKO cortices stained for PCDH19 (green) and counter-stained with DAPI (blue). High-magnification images of the superficial and deep layers (broken lines) are shown to the right. Note the decrease in PCDH19 expression in the *Tbr2* mutant cortex compared with the control. Scale bars: 100 μm and 10 μm. **f,** Quantification of the relative PCDH19 staining intensity in the control and *Tbr2* cKO cortices (n = 3 for each genotype). Data are presented as mean ± SEM (**$P < 0.01$, ***$P < 0.001$; unpaired Student's t-test).

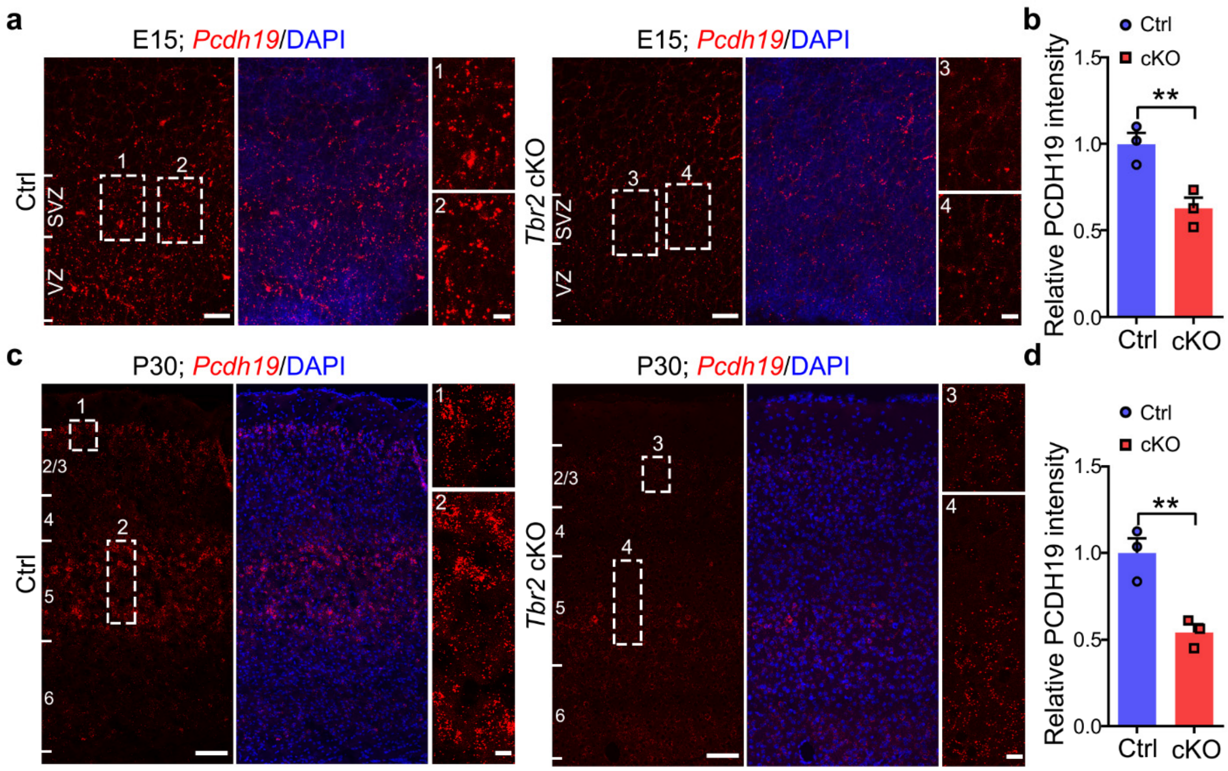

Supplementary Fig. 13: TBR2 removal leads to a reduction in *Pcdh19* expression revealed by FISH. a, Representative confocal images of fluorescence in situ hybridization (FISH) for *Pcdh19* (red) in E15 Ctrl and *Tbr2* cKO SVZ/VZ regions. Note the decrease in *Pcdh19* expression in the *Tbr2* cKO SVZ/VZ compared with Ctrl. High-magnification images (broken lines) are shown to the right. Scale bars: 30 µm and 10 µm. **b,** Quantification of the relative *Pcdh19* FISH intensity in Ctrl and *Tbr2* cKO SVZ/VZ (n = 3 for each genotype). **c,** Representative confocal images of FISH for *Pcdh19* (red) in P30 Ctrl and *Tbr2* cKO cortices. High-magnification images (broken lines) are shown to the right. Note the decrease in *Pcdh19* expression in the *Tbr2* cKO cortex compared with Ctrl. Scale bars: 100 µm and 10 µm. **d,** Quantification of the relative *Pcdh19* fluorescence intensity in Ctrl and *Tbr2* cKO cortices (n = 3 for each genotype). Data are presented as mean ± SEM (**P < 0.01, unpaired Student's t-test).

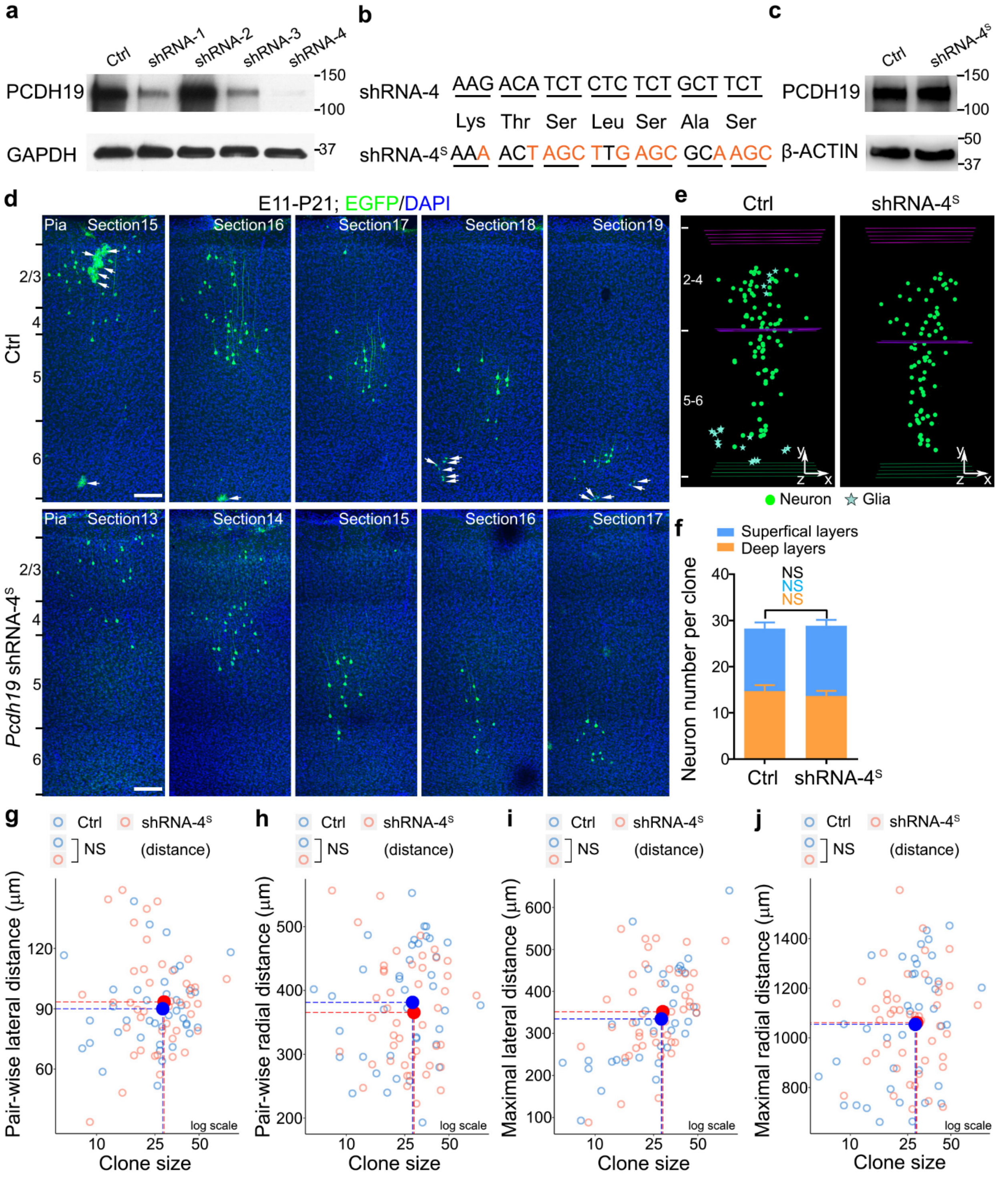

Supplementary Fig. 14: Specificity of *Pcdh19* shRNA-4 in suppressing PCDH19 expression. **a,** Efficacy of Pcdh19 shRNA 1-4 in suppressing PCDH19 expression. Control or *Pcdh19* shRNA plasmid 1-4 was co-transfected with *Pcdh19* expression plasmid into HEK 293T cells. PCDH19 level was analyzed by western blotting ~3 days later. GAPDH was used as a control. **b,** *Pcdh19* shRNA-4 targeting sequence and *Pcdh19* shRNA-4 mutant/scrambled (*Pcdh19* shRNA-

4^S) sequence. **c,** shRNA-4^S failed to suppress Pcdh19 expression confirmed by western analysis in HEK293T cells. **d,** Representative confocal images of P21 excitatory neuron clones expressing EGFP and Ctrl (top) or *Pcdh19* shRNA-4^S (bottom). Scale bar: 100 µm. **e,** 3D reconstruction images of Ctrl and *Pcdh19* shRNA-4^S clones shown in d. **f,** Quantification of the number of neurons in individual clones expressing Ctrl (n = 39) or *Pcdh19* shRNA-4^S (n = 45). **g-j,** Quantification of the pair-wise (g,h) and maximal (i,j) lateral and radial distances between neurons in Ctrl (n = 39) and *Pcdh19* shRNA-4^S (n = 45) expressing clones. Data are presented as mean ± SEM (NS, not significant; unpaired Student's t-test).

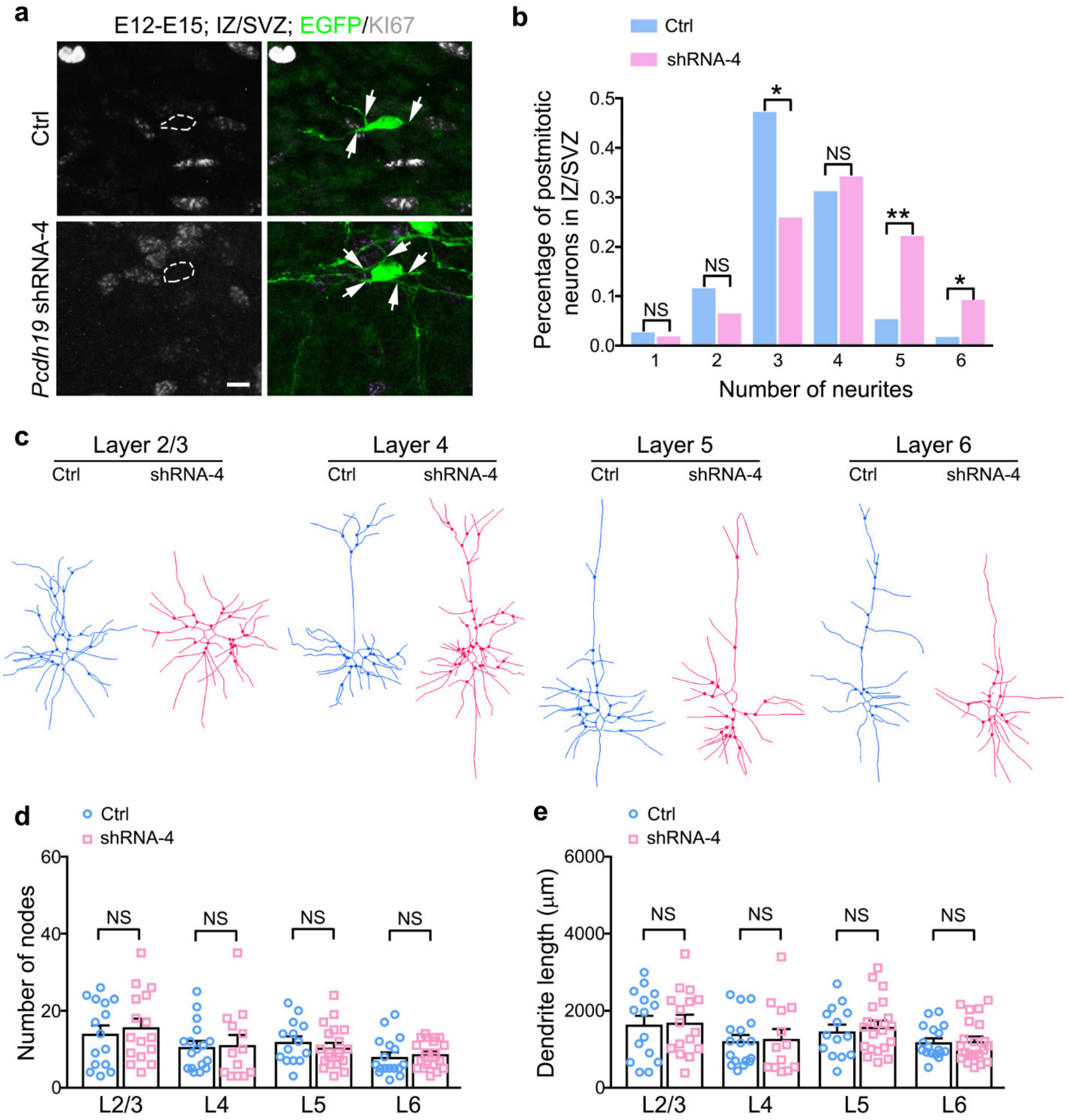

Supplementary Fig. 15: Suppression of PCDH19 expression leads to a transient increase in neurite number of new-born neurons. a, Confocal images of representative E15 Ctrl and PCDH19 knockdown clones labeled by low-titer retrovirus at E12 and stained for KI67 (white). Note the neurons expressing EGFP (green) but not KI67. Scale bar: 10 μm. **b,** Quantification of the percentage of neurons possessing different numbers of neurites in the Ctrl and PCDH19 knockdown clones (Ctrl, *n* = 112 neurons from 49 clones; shRNA-4, *n* = 108 neurons from 57 clones). Data are presented as mean ± SEM (**P* < 0.05; ***P* < 0.01; NS, not significant; chi-square test). **c,** Representative reconstructed morphologies of Ctrl and PCDH19 knockdown neurons in different layers. **d,e,** Quantification of the neurite branch number (d) and length (e) of Ctrl and PCDH19 knockdown neurons in different layers (L2/3, Ctrl, *n* = 15; Mut, *n* = 16; L4,

Ctrl, *n* = 17; Mut, *n* = 13; L5, Ctrl, *n* = 14; Mut, *n* = 20; L6, Ctrl, *n* = 17; Mut, *n* = 21). Data are presented as mean ± SEM (NS, not significant; unpaired Student's t-test).

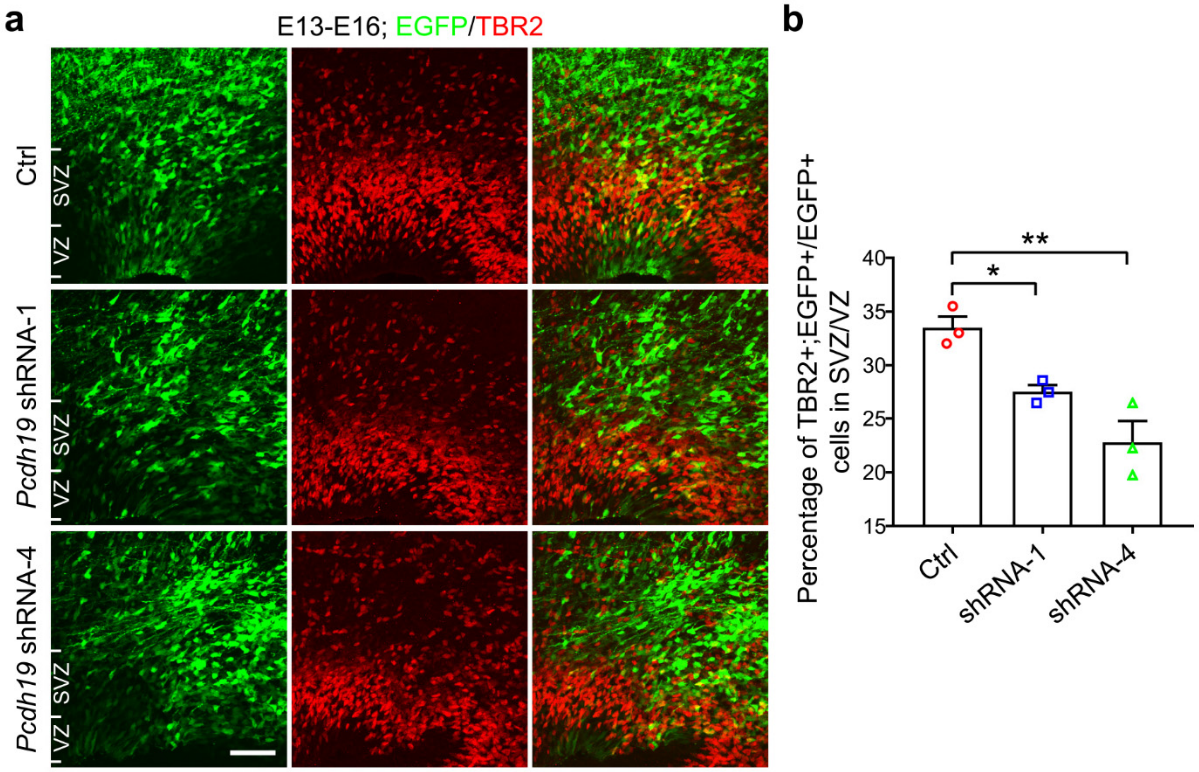

Supplementary Fig. 16: Suppression of PCDH19 expression leads to a loss of TBR2$^+$ IPs. a, Confocal images of representative E16 EGFP/Ctrl and EGFP/*Pcdh19* shRNA-expressing (green) cortices electroporated at E13 and stained for TBR2 (red). Scale bar: 50 µm. **b,** Quantification of the percentage of EGFP$^+$;TBR2$^+$/EGFP$^+$ cells in the SVZ/VZ (Ctrl, n = 3; shRNA-1, n = 3; shRNA-4, n = 3). Data are presented as mean ± SEM (*$P < 0.05$; **$P < 0.01$; unpaired Student's t-test).

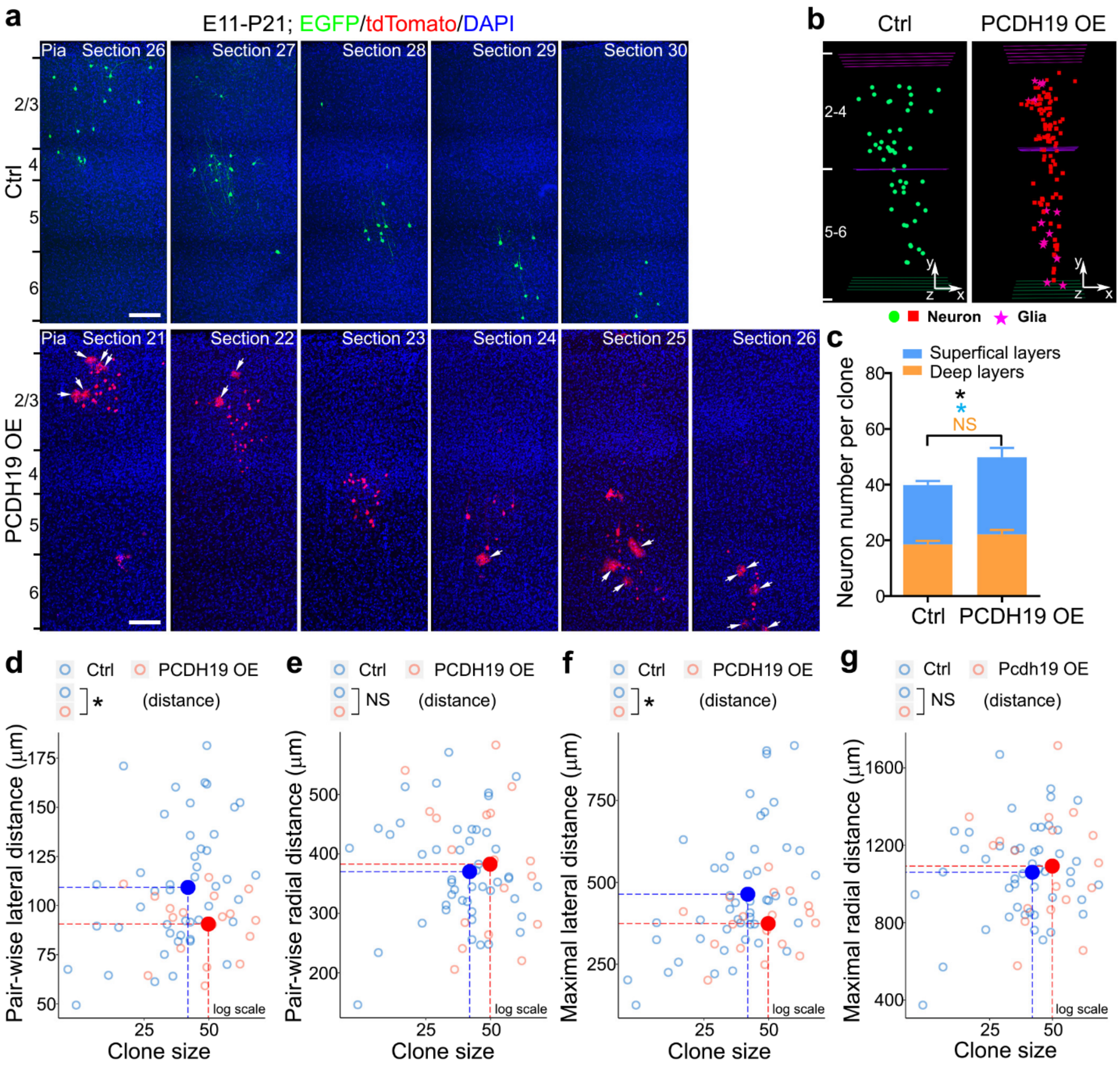

Supplementary Fig. 17: PCDH19 overexpression leads to an increase in neuronal output of individual RGPs and lateral clustering of clonally related excitatory neurons. a, Representative confocal images of P21 Ctrl/EGFP (top, green) or PCDH19/tdTomato overexpression (bottom, red) clones. Arrows indicate glial cells. Scale bars: 100 µm. **b,** 3D reconstruction images of Ctrl and PCDH19 overexpression clones shown in a. **c,** Quantification of the number of neurons in Ctrl (n = 48) or PCDH19overexpression (n = 18) clones. **d,e,** Quantification of the pair-wise lateral and radial distances between neurons in Ctrl (n = 48) or PCDH19overexpression (n = 18) clones. **f,g,** Quantification of the maximal lateral and radial distances between neurons in Ctrl (n = 48) or PCDH19 overexpression (n = 18) clones. Data are presented as mean ± SEM (*$P < 0.05$; NS, not significant; unpaired Student's t-test).

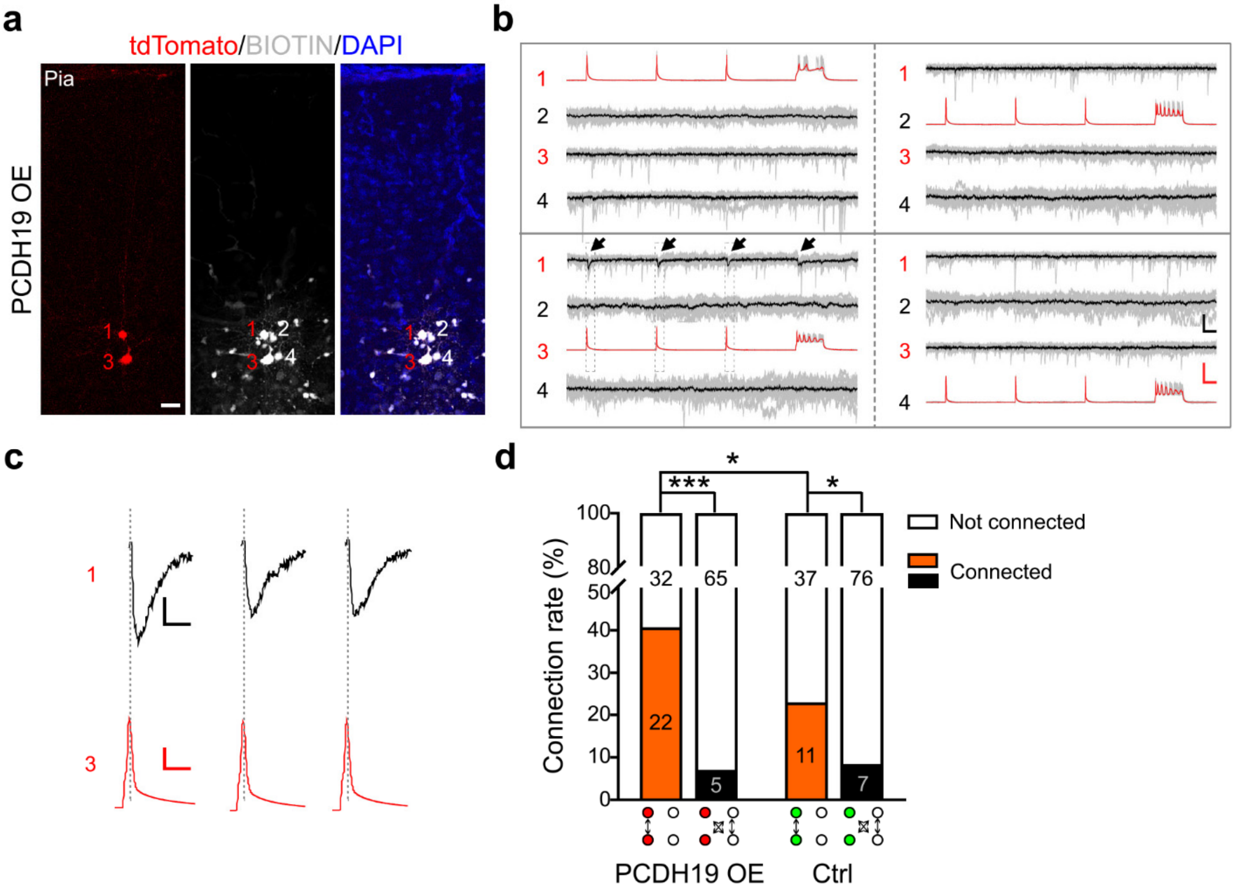

Supplementary Fig. 18: PCDH19 overexpression leads to an increase in synaptic connectivity between clonally related cortical excitatory neurons. a, Representative confocal images of two radially situated PCDH19/tdTomato-expressing clonally related excitatory neurons (1 and 3, red) labeled at E12 and two adjacent unlabeled non-clonally related excitatory neurons (2 and 4). Scale bars: 50 µm. **b,** Example traces of four excitatory neurons recorded in a. Brief and long duration depolarizing currents were injected into one of the four neurons to elicit action potentials (presynaptic, red) and postsynaptic responses were monitored in the other three neurons. Individual traces are shown in grey and average traces are shown in black. Arrows indicate the reliable postsynaptic responses in PCDH19/tdTomato-expressing excitatory neuron 1 elicited by the presynaptic action potentials in PCDH19/tdTomato-expressing excitatory neuron 3. Scale bars: 100 mV (red), 20 pA (black), and 200 msec. **c,** Zoom-in traces of the presynaptic action potentials and postsynaptic responses between PCDH19/tdTomato-expressing clonally related excitatory neurons 1 and 3 in b. Scale bars: 25 mV (red), 10 pA (black), and 25 msec. **d,** Summary of the frequency of chemical synaptic connections between Ctrl (green) or PCDH19/tdTomato-expressing (red) clonally related excitatory neurons and nearby non-clonally related excitatory neurons. The numbers of recorded pairs are shown in the bar graph. (*$P < 0.05$; ***$P < 0.001$; NS, not significant; chi-square test).

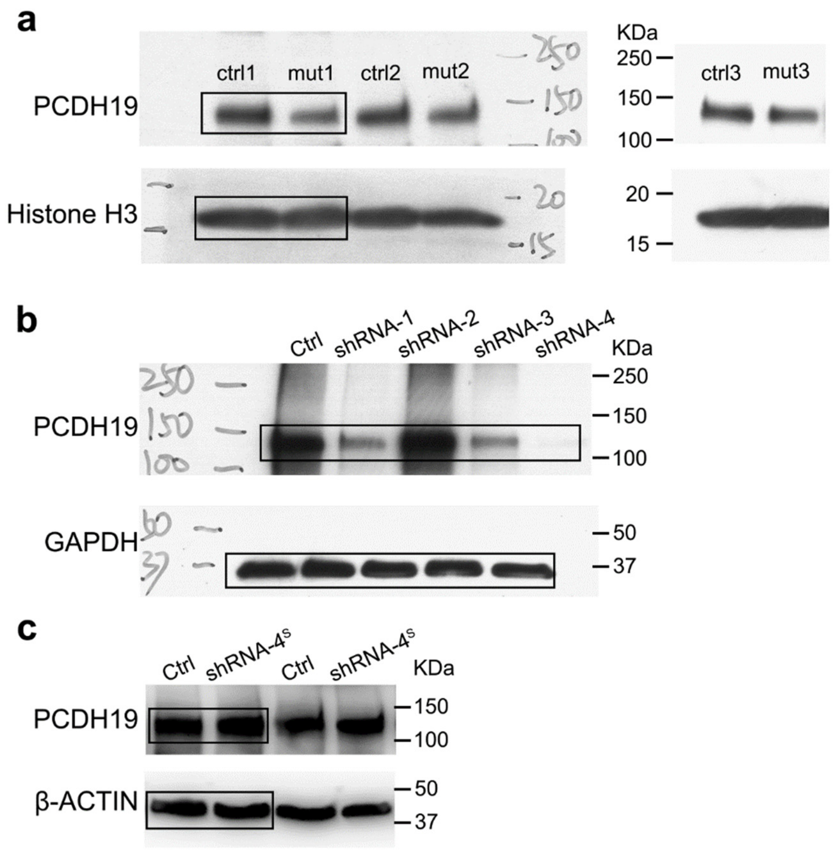

Supplementary Fig. 19: Uncropped scans of blots with size marker indications. a, Uncropped blots of Fig. 4h. **b,c,** Uncropped blots of supplementary Figs. 14a and 14c respectively. Edited blots were marked by black rectangles.
