# Supplementary material for: TBR2 coordinates neurogenesis expansion and precise microcircuit organization via Protocadherin 19 in the mammalian cortex
Source: Nat Commun. 2019 Sep 2;10:3946. doi: 10.1038/s41467-019-11854-x (PMC6718393; doi:10.1038/s41467-019-11854-x)
Supplement: Supplementary file 2 — Description of Additional Supplementary Files [file 41467_2019_11854_MOESM2_ESM.docx]

**Description of Additional Supplementary Files**

**File Name:** Supplementary Data 1

**Description:** Overrepresented/Enriched Gene Ontology (GO) terms associated with the set of genes that are differentially expressed between the Ctrl and *Tbr2* mutant IPs.

**File Name:** Supplementary Data 2

**Description:** Sets of differentially regulated genes in the *Tbr2* mutant IPs compared with Ctrl at E13 and E16.

**File Name:** Supplementary Data 3

**Description:** The list and sequences of primers used for *Pcdh19* shRNA, qPCR, ChIP-qPCR, and luciferase assays.
