# Supplementary material for: TBR2 coordinates neurogenesis expansion and precise microcircuit organization via Protocadherin 19 in the mammalian cortex
Source: Nat Commun. 2019 Sep 2;10:3946. doi: 10.1038/s41467-019-11854-x (PMC6718393; doi:10.1038/s41467-019-11854-x)
Supplement: Supplementary file 6 — Reporting Summary [file 41467_2019_11854_MOESM6_ESM.pdf]

nature research

Corresponding author(s): Song-Hai Shi

Last updated by author(s): Jul 30, 2019

Reporting Summary

Nature Research wishes to improve the reproducibility of the work that we publish. This form provides structure for consistency and transparency in reporting. For further information on Nature Research policies, see Authors & Referees and the Editorial Policy Checklist.

Statistics

For all statistical analyses, confirm that the following items are present in the figure legend, table legend, main text, or Methods section.

n/a	Confirmed	
☐	☒	The exact sample size (*n*) for each experimental group/condition, given as a discrete number and unit of measurement
☐	☒	A statement on whether measurements were taken from distinct samples or whether the same sample was measured repeatedly
☐	☒	The statistical test(s) used AND whether they are one- or two-sided<br>*Only common tests should be described solely by name; describe more complex techniques in the Methods section.*
☐	☒	A description of all covariates tested
☐	☒	A description of any assumptions or corrections, such as tests of normality and adjustment for multiple comparisons
☐	☒	A full description of the statistical parameters including central tendency (e.g. means) or other basic estimates (e.g. regression coefficient) AND variation (e.g. standard deviation) or associated estimates of uncertainty (e.g. confidence intervals)
☐	☒	For null hypothesis testing, the test statistic (e.g. *F*, *t*, *r*) with confidence intervals, effect sizes, degrees of freedom and *P* value noted<br>*Give P values as exact values whenever suitable.*
☒	☐	For Bayesian analysis, information on the choice of priors and Markov chain Monte Carlo settings
☒	☐	For hierarchical and complex designs, identification of the appropriate level for tests and full reporting of outcomes
☒	☐	Estimates of effect sizes (e.g. Cohen's *d*, Pearson's *r*), indicating how they were calculated

Our web collection on statistics for biologists contains articles on many of the points above.

Software and code

Policy information about availability of computer code

Data collection	Confocal microscope (FV1000, Olympus), Neurolucida and Stereo Investigator (MBF Bioscience), Axon Multiclamp 700B amplifier.
Data analysis	FluoView (Olympus), Volocity (ImproVision), Image J (NIH), and Photoshop (Adobe), Neurolucida and Stereo Investigator (MBF Bioscience), MATLAB (MathWorks), R-Studio (RStudio), pCLAMP 10 software.

For manuscripts utilizing custom algorithms or software that are central to the research but not yet described in published literature, software must be made available to editors/reviewers. We strongly encourage code deposition in a community repository (e.g. GitHub). See the Nature Research guidelines for submitting code & software for further information.

Data

Policy information about availability of data

All manuscripts must include a data availability statement. This statement should provide the following information, where applicable:

- Accession codes, unique identifiers, or web links for publicly available datasets
- A list of figures that have associated raw data
- A description of any restrictions on data availability

Microarray datasets are available at the GEO with the accession number: GSE121180 and GSE45450. The other data that support the findings of this study are available from the corresponding author upon request.

Field-specific reporting

Please select the one below that is the best fit for your research. If you are not sure, read the appropriate sections before making your selection.

☒ Life sciences ☐ Behavioural & social sciences ☐ Ecological, evolutionary & environmental sciences

For a reference copy of the document with all sections, see nature.com/documents/nr-reporting-summary-flat.pdf

Life sciences study design

All studies must disclose on these points even when the disclosure is negative.

Sample size	Sample size was estimated based on existing published research.
Data exclusions	No data was excluded.
Replication	All replications were successful (at least 3 times).
Randomization	For all animal experiments, mice were allocated randomly after genotyping.
Blinding	Data was analyzed by researchers blind to genotypes.

Reporting for specific materials, systems and methods

We require information from authors about some types of materials, experimental systems and methods used in many studies. Here, indicate whether each material, system or method listed is relevant to your study. If you are not sure if a list item applies to your research, read the appropriate section before selecting a response.

Materials & experimental systems

n/a	Involved in the study	
☐	☒	Antibodies
☐	☒	Eukaryotic cell lines
☒	☐	Palaeontology
☐	☒	Animals and other organisms
☒	☐	Human research participants
☒	☐	Clinical data

Methods

n/a	Involved in the study	
☒	☐	ChIP-seq
☒	☐	Flow cytometry
☒	☐	MRI-based neuroimaging

Antibodies

Antibodies used	The primary antibodies used in Immunohistochemistry were chicken anti-GFP (Aves lab, GFP-1020, 1:1000), rabbit anti-RFP (Rockland, 600-401-379, 1:1000), rabbit anti-PCDH19 (Thermo, PA5-55648, 1:50), rabbit anti-S100 (DAKO, Z0311, 1:500), rabbit anti-OLIG2 (Millipore, ab9610, 1:500), rabbit anti-Histone H3 (CST, 4499, 1:1000), rabbit anti-Cleaved Caspase-3 (CST, 9664, 1:500), rabbit anti-CUX1 (Santa Cruz, sc-13024, 1:100), rat anti-KI67 (Thermo, 14-5698-82, 1:100), rat anti-TBR2 (Thermo, 14-4875-82), rat anti-CTIP2 (Abcam, ab18465, 1:500), goat anti-FOXP2 (Santa Cruz, sc-21069, 1:100), goat anti-BRN2 (Santa Cruz, sc-6029, 1:200), and mouse NEUN (Millipore, MAB377, 1:100).The secondary antibodies were used: goat anti-chicken IgY (H+L) 488 (Thermo, A11039, 1:100), donkey anti-rabbit IgG (H+L) Cy3 (Jackson ImmunoResearch, 711-165-152, 1:1000), goat anti-rat IgG (H+L) 647 (Thermo, A21247, 1:1000), donkey anti-rat IgG (H+L) Cy3 (Jackson ImmunoResearch, 712-165-150, 1:1000), donkey anti-rat IgG (H+L) 488 (Thermo, A21208, 1:1000), donkey anti-goat IgG (H+L) 488 (Thermo, A32814, 1:1000), goat anti-mouse IgG1 488 (Thermo, A21121, 1:1000). The primary antibodies used in western blotting were rabbit anti-PCDH19 (Millipore, ABT318, 1:2000), rabbit anti-GAPDH (Santa Cruz, sc-32233, 1:1000), rabbit anti-Histone H3 (CST, 4499, 1:5000), and mouse anti-β-ACTIN (Proteintech Group, 60008-1, 1:5000).
Validation	All antibodies were obtained from commercial vendors. The antibodies were purchased based on previous validation from publications from highly sources.

Eukaryotic cell lines

Policy information about cell lines

Cell line source(s)	HEK 293T cell line was maintained in the lab and published in previous work (Yu et al, Nature, 2009).
Authentication	We used functional assay of virus generation from the HEK 293T cells. HEK 293T cells were also used for western blot experiments.

Mycoplasma contamination	HEK 293T cell line used in this study was verified to be mycoplasma free.
Commonly misidentified lines (See ICLAC register)	No commonly misidentified cell line was used.

Animals and other organisms

Policy information about studies involving animals; ARRIVE guidelines recommended for reporting animal research

Laboratory animals	6-8 wk Emx1-CreERT2;MADM-11GT/GT;Tbr2fl/+; 6-8 wk MADM-11TG/TG;Tbr2fl/fl; 6-8 wk Tbr2fl/fl; 6-8 wk Emx1-Cre; both female and male were used.
Wild animals	None used
Field-collected samples	None used
Ethics oversight	MSKCC animal committees

Note that full information on the approval of the study protocol must also be provided in the manuscript.
